# Critical consideration towards broad consent by patient experts: results of a semi-structured interview study on the secondary use of medical data

**DOI:** 10.1186/s12910-025-01326-x

**Published:** 2025-11-18

**Authors:** Henk Jasper van Gils-Schmidt, Jan Hinrichsen, Silke Schicktanz, Sabine Wöhlke

**Affiliations:** 1 Hamburg University of Applied Sciences, Hamburg, Germany; 2 Department of Medical Ethics and History of Medicine, University Medical Center Göttingen, Göttingen, Germany; 3 Faculty of Health, Hamburg University of Applied Sciences, Hamburg, Germany

**Keywords:** Broad Consent, Secondary research use, Patient attitudes, Semi-structured interview study, Dynamic consent

## Abstract

**Background:**

Some would argue that there is a moral obligation to make the personal health data stored in databases and biobanks available for secondary research use. Yet, it is unclear what ways to gain consent to use the stored data for secondary research purposes are both effective and respectful of the patient’s autonomy. One prominent example under discussion is broad consent. As a form of consent in which specific study objectives are not defined, it is seen by many as an efficient alternative to the established consent of participants. Research on subjects' attitudes towards and their discursive reflections on broad consent is, however, limited. With our study, we aimed to gain deeper insights into the views and (normative) attitudes towards broad consent by members and representatives of patient organizations.

**Methods:**

Semi-structured interviews were conducted with members (*N* = 13) and representatives (*N* = 9) of German patient organizations. Subsequently, we evaluated the material using content analysis.

**Results:**

The results initially indicate a general agreement with broad consent among members and representatives of patient organizations. In contrast to the results of some existing studies on broad consent, our analysis reveals limitations in this regard: Positive assessments relate less to broad consent in particular, but rather to overarching regulations on secondary data use that deviate from broad consent. Broad consent is criticized for exactly what it was originally intended to regulate: reduced flow of information, lack of a concrete and communicated research objective, and coverage of (too) long periods of time. The interviewees often formulated specific ideas and wishes about appropriate procedures for consenting to secondary data use, i.e., specific governance structures, thus (implicitly) conceptualizing "informed consent" as the gold standard of consent procedures.

**Conclusions:**

The interviewees consider the provision of data for secondary use to be important for the improvement of treatment methods, freedom of research, and ethical considerations such as reciprocity. However, these values are linked to conditions in the stakeholders' considerations and thus appear less absolute and universal than conditional and situated. Qualitative, empirical-ethical research can make this inherent complexity of ethical attitudes of stakeholders as negotiation processes tangible and fruitful for medical ethical practice.

**Supplementary Information:**

The online version contains supplementary material available at 10.1186/s12910-025-01326-x.

## Background

In health research, large databases and biobanks gain evermore in importance, especially against the background of the digital transformation of research through AI- and algorithms-based research innovations [1]. Such research, which delves huge and complex data sets, discovers patterns that the human mind fails to observe. Considering the health domain, some would argue that there is a moral obligation to use the personal health data gathered in databases and biobanks in research and clinics if people are admitted, as this furthers our understanding of diseases significantly and brings us closer to better diagnostics and more effective treatment options [47]. The use of health data for research that was either stored for nonresearch purposes or for a research purpose other than the current study is called “secondary research use” [25].^3^ Up to now, such secondary use relied on a renewed informed consent by the person whose health data it is (alternatively a waiver by the proper authorities, as it was the case in the USA [25], or a different legal justification without consent can be used, which is only used in rare cases, though). Obtaining such renewed consent is, however, immensely cumbersome time- and money-wise [9], and runs the risk of data loss, when consent is not renewed, resulting also in a quality loss of the data set.

Around the globe, many initiatives are undertaken to integrate health data from different sources and technical and legal infrastructures are being set up for the secondary use of health care data [45]. In Germany, the Medical Informatics Initiative develops the necessary prerequisites for linking and sharing routine clinical care data in Germany to become available for research [27, 40], as does the NFDI4Health initiative for health data bases generated in context of clinical, epidemiological and public health research projects [13, 31]. Based on the Act on the Use of Secondary Data, the Finnish agency FINDATA provides researchers with access to social and health data of the Finnish population in a pseudonymized form and a protected virtual environment [29]. Similarly, Australia has created the possibility for publishing pseudonymized data from an electronic health record, MyHealthRecord, for research purposes [3]. On the political level, the will to unlock the unused potential of medical data is further expressed in the European Health Data Space proposed by the European Commission as well as the German *Gesundheitdatennutzungsgesetz* (Engl.: Health Data Use Law; came into effect on 26.03.2024). Both political actions define a data use practice consisting of shared standards of data storage and use, infrastructures, and a governance framework that builds in checks and balances. Within the U.S. context, the federal regulatory provisions have recently replaced the required informed consent for participants in research with human subjects funded on the federal level with a broad consent [42].

Against this background, many perceive broad consent as an efficient alternative for the traditional “gold standard” of informed consent. Qualitative, in-depth, and descriptive research in this area is limited (see below), especially in regards the reflections of members and representatives of patient organizations (PO). Based on the empirical-ethical PANDORA research project, which inquires into the ethical challenges of digitalization in the health sector and secondary data, the present interview study was conducted with members and representatives of PO that mostly represent rare, chronic diseases. Our study aims to gain deeper insights into the complex field of attitudes and opinions of those who sign broad consent forms that reaches beyond “approval” or “rejection”-attitudes towards broad consent by analysing the reflection of members and representatives of PO on the topic of broad consent for health data.

As simultaneously experts – as we frame their engagement (see below) – and affected persons of consent procedures, specifically these stakeholders provide insights of extraordinary value for a deeper understanding of the ethical challenges of (broad) consent procedures. While the data from such studies is context-specific and not statistically generalizable, employing the expertise of members and representatives of PO in qualitative research yields profound results for the debate on ethically acceptable regulation and governance of secondary data use.

After laying out the theoretical background of the discussion of broad consent and our research methodology, we present the results from our empirical-ethical interview study along three interrelating fields: affirmative attituded towards secondary date usage; critical considerations of broad consent procedures; and alternative suggestions for the regulation of consent for secondary data usage. We will discuss our findings against the current debate on broad consent.

### Theoretical background

In contrast to informed consent in which the research purpose is made explicit, with broad consent individuals consent with the use of their medical data or biolocal material for future research of which the study aims are described solely in general termsand thus stay undefined. The rationale for this is the expectation that the analysis of the data/material lead to new research aims that can be studied with the same material. Broad consent allows this. In other words, broad consent provides the legal basis for the secondary use of data [25].

Yet, broad consent is not the same as “blanket consent,” which implies unrestricted future use without any limitations. Broad consent typically includes clear ethical and legal boundaries for secondary use by placing limits on the types of research permitted (e.g., exclusion of commercial use or genetic studies) and by establishing procedural safeguards, such as governance structures involving ethics committee review or the right to withdraw consent (cf. [15]).

Simultaneously, broad consent introduces a different time dimension [48]. Informed consent is given within a specific set of regulations, laws as well as scientific values and practices that hold at the time of the consent. However, broad consent widens the time dimension immensely (up to 30 years in the European context). Within this time, regulations, laws as well as research values and practices regarding data use can change radically.

Within the scientific literature, voices for and against broad consent are present. It has been argued that broad consent violates the requirement of thorough information on the side of the patients [16, 17, 28, 34] and the hard fought-for right to being informed before becoming a research participant, i.e., the right to informed consent based on the respect of persons is undermined [41, 46]. It is feared that broad consent could become “a sieve, not a safeguard” [4]. Furthermore, without any or much information on the aims of data use, risk-assessment for participants is difficult, if not impossible [50]. Due to changed (social) values the stored data might be used for purposes the consenting persons may have, based on their values, never agreed to.

Proponents have pointed out the merits of a broad consent, such as its great value for public health [5], the unknowns involved in many types of (medical) research [15], respect for the individual`s autonomy in deciding to participate in research or not [43]. Some also have pointed out the conceptual impossibility of informed consent in the context of biobanking which can easily be transferred to databases [8, 21]. The underlying idea is that the purpose of a database is to enable future research based on information that is not yet available. This makes informed consent impossible and creates the ethical imperative to employ other forms of consent, such as a broad consent (or, e.g., blanket, tiered, meta consent,see discussion.

Research across the globe finds that patients and the general public show positive attitudes towards broad consent [18, 26, 30, 44, 50] Despite such evidence for positive appraisal of broad consent, it is important to note that support for broad consent is not universal. Some studies have shown that if pharmaceutical companies gained access to data the willingness to share data was lower [10, 14] and single country-specific studies showed a lower willingness of the general public (e.g., from Japan, see [32]). For the German context in which our study took place, quantitative studies show a strong willingness for broad consent for secondary data use as well as an approval of discontinuing the informed consent practice [22, 37–39]. However, most of these studies rely on standardized questionnaires and do not allow for a discursive engagement with participants.

While findings of these studies reflect general attitudes, they do not distinguish between the views of patients and the broader public. In the context of secondary use of large-scale health data, both perspectives are relevant—because they may be shaped by different experiences and expectations. Patients, as direct contributors of data and experts of experience (see our discussion below), may hold specific ethical concerns regarding the structure and governance of broad consent policies. Our study focuses specifically on these patients’ (ethical) considerations regarding the structure and design of broad consent policies, aiming to complement existing survey-based research with a more nuanced, qualitative understanding of the views of those most directly affected.

## Methods

### Study context, design and target group

We conducted a semi-structured interview study in context of the research network PANDORA. PANDORA investigates the role of PO in the digital transformation of the health system as well as their view on different aspects of the digitalization process in health research. To report our methodology, we use the COREQ Checklist (see additional file 1).

For this study, we invited members and representatives of PO in Germany to share their views and attitudes on the topic of broad consent as alternative consent model for informed consent. We choose this target group, as we take members and representatives of patient organizations to be “experts of experience” with a specific and outstanding position on the field of patient representation [35, 36]. As both experts and affected person, they provide profound insights in health politics. We interviewed in total *N* = 22 participants with whom no prior relationship existed (see Table 1 for demographics). Representatives (*N* = 9) could either be volunteers in or employed by the PO and had to be in a leadership role. Our target was to include diverse PO regarding size, thematic focus and represented disease. Members were recruited from two different PO, Mukoviszidose e. V. and PRO RETINA Deutschland e. V. (*N* = 13). Both organizations have their own patient registry that they make available for research, which was a prerequisite for us due to the focus of our study, and members had to have made a conscious decision whether to give their informed consent to share their medical data for research purposes through the registry (Table 2).

**Table 1:** Characteristics of participants, including interview length

	**Gender**	**Age (average)**	**Highest degree**	**Length interview (average)**
Members	5 Female/8 Male	47,6 years (range: 18–76)	1 PhD 9 higher education 2 Vocational education 1 no degree	~ 54 Minutes
Represen-tatives	5 Female/4 Male	53,3 years (range: 31–72)	2 PhD 7 higher education	~ 57 Minutes

**Table 2:** Characteristics of patient organizations (*n* = 9)

Characteristic		Patient organizations, n (%)
Size (members), n		
100–500	1 (11,1)	
501–1000	2 (22,2)	
1001–10,000	2 (22,2)	
10,001–100,000	0 (0)	
> 100,000	1 (11,1)	
N/A^a^ (umbrella organization)	3 (33,3)	
Geographic scope		
Regional	1 (11,1)	
National	8 (88,9)	
Disease area		
Chronic disease	3 (33,3)	
Rare chronic disease	3 (33,3)	
Chronic disease and disability (umbrella organizations)	3 (33,3)	

^a^N/A: not available

### Recruitment strategy and data collection

To recruit the members, a manual search via PO websites was executed to identify POs that met the criteria (HJvGS). In a purposive recruitment strategy, contact was established with four POs of which three forwarded the invitation to their members. Representatives were identified based on a convenience strategy through targeted internet research, an invitation to all member organizations of the umbrella organization B.A.G. Selbsthilfe e. V., and direct contacts with PO (HJvGS). In total, contact was established with 30 potential interviewees, 8 dropped out for unknown reasons.

The interviews were conducted and audiorecorded between October 2022 and March 2023 by telephone (HJvGS). The conductor, a male senior research fellow, had a background in philosophy (PhD), empirical medical ethics and psychology. He developed a semi-structured interview guide for each target group following the methods of qualitative research [19]. Feedback was first given by SW and SiS as well as by the PANDORA patient advisory board. Subsequently the guides were pretested. The guidelines had different questions but addressed two topics: 1) positive and negative aspects of digitalization in medical research and health care; 2) data use and the role of patients (see additional file 2 for the interview guide; Question 10 in the guideline for PO members; Questions 11 & 12 in the quidline for PO representative). For the present article we focus on broad consent. The questions on broad consent were started by asking after the understanding of broad consent and zooming in on the definition provided in the interview guide to ensure all participants spoke to the same construct (“*Broad consent means that patients are asked to give their consent to a wide range of future research projects, the content of which cannot yet be foreseen at the time of consent*”). Before an appointment for the interview was made, an informed consent form to participate in this study was signed by all the participants. At the start of each interview, it was ensured that only the conductor and interviewee were present and general information on the aim of the study and the role of the conductor was given. No repeat interviews were carried out and field notes were only used to structure the interviews during the call.

### Data analysis

The audio files were transcribed verbatim, pseudonymized, and analyzed according to the Qualitative Content Analysis method [23]. The analysis was done in MAXQDA. Transcripts were not returned to the participants. Two coding guides were developed deductively, including code names, rules for coding, and anchor examples (see additional file 3). Two coders, HJvGS and research student assistant Stella Kaltenbach (SK), first summarized the transcripts and, second, made memos regarding the main research questions. To establish intercoder agreement and to test the coding guide, four transcripts were coded independently by HJvGS and SK and the preliminary coding guide was slightly adapted afterwards. Subsequently, all material was coded. Finally, HJvGS compiled all text passages coded with the same category, HJvGS and JH executed an analysis of the data to subtract overarching themes, JH selected exemplary quotes for this paper (see results section) and translated them using the Deepl.com translation software for an initial translatation that was then edited manually and adjusted the narrative style of the interview (see additional file 4 for the quotes in the German original). No feedback on the findings by the participants was provided.

### Limitation of the sample

Only patients whose organization operates its own data registry and had direct experience with consent procedures, were included. This raises the question of possible differences in the perception of broad consent by patients without such a specific background. Given a high trust in the PO and towards the patient registry in general, we hold that the bias would rather appear towards a positive attitude regarding broad consent. This limiting factor does not hold for the representatives that participated. What is more, data from qualitative studies with small samples is context-specific and not readily statistically generalizable.

## Results

Our results show a complex field of attitudes and opinions of our interlocutors, reaching beyond “approval” or “rejection”-attitudes towards broad consent, instead indicating a conditional relation to broad consent in which favorsome positions go hand in hand with critical stances and are often tied to explicitly phrased preconditions. The results are structured along three main axes: First, the representatives’ and members’ approval of and/or positive attitudes towards consent procedures enabling secondary data usage. Second, the interviewees’ negative attitudes/inhibitions and their reasons for problematizing broad consent. Third, alternative suggestions, proposals for improvement, and general wishes of the interviewed towards procedures regulating secondary use of health data.

### Positive aspects: positive impact on research and reciprocity

Six representatives and six members of PO phrased affirmative positions towards broad consent in context of secondary data use. More representatives than members emphasized the need for analyzing large quantities of data for political decision making, underpinning the necessity of evidence-based (health) politics. Against this background, it would, to some of our interviewees, even be unethical to not collect (and therefore re-use) health data:

“It's good that you can [use existing health data for research] and it would be unethical in my view if you didn't collect this data today in order to make decisions in the future and be able to derive decisions from it.” (Representative_1)

Broad consent is thus favored for its lean bureaucratic nature and low financial burden, while it is also viewed as an empowering tool for patients opposing to paternalize them.

By and large, our interviewees emphasized datasharing as a means and act of “giving something back” and thus of reciprocity::

“The only way I can give something back is that at least, especially with such an unknown illness, I am prepared to make myself available, as long as it doesn't hurt and as long as it doesn't kill me.” (Representative_2)

“And in essence, I also think that the individual must take a back seat to the community, so to speak, because ultimately it is the community as a whole or society that counts and not the individual in essence.” (Representative_3)

The reasons for these acts of reciprocity are found in the conviction that the possibility to access data for secondary use on a large scale is beneficial to the freedom and quality of research: Donating data, in this perspective, serves the production of novel and crucial scientific facts, especially in regards rare diseases:

“The advantage is, absolutely for our rare diseases, which always fall behind somehow. (...) Because they are so rare, it is very important to gather as much data as possible from the broad field and not to keep asking for consent again and again, but, yes. And finally, the rare diseases in particular. There is still nothing. There is still no real basic drug, still only symptoms are being treated. (…) There are many, many important research questions that simply reduce the process and therefore also the costs of research.” (Representative_4)

Thus, making available their medical data directly improves treatment methods, especially in the view of members.

“I have to be honest and say that, if it is in relation to [the disease I have], I would release my data. (…) I have no problems with that at all, [so that] better data on care can be developed [...].” (Member_1)

Simultaneously, the donation of data was conceptualized as a form of exchange: the scientific community was held to offer a (non-monetary) compensation for donated data:

“[...] I think that a system should be established so that if you have to give up your data permanently, you get something in return. And I don't mean fifty euros per data set or anything like that, but that there should at least be transparency about (...) where exactly my data ends up in which research project, what research is done with it and what comes out of it, where it is published and where I get the information, etc.” (Representative_3)

### Negative aspects: temporal dimension & lacking objectives

Notwithstanding their positive attitudes, the interviewees simultaneously painted a critical picture appearing much more layered, nuanced and differentiated. The topic of broad consent served as a platform for critique regarding adequate data security and the problems concerned in the sharing of health data in general, thereby going beyond narrow conceptualizations of broad consent.

The reasons for the critical attitudes are manifold and, in this multidimensionality, evenly distributed between representatives and members alike. A risk of expropriation of data on the side of the patients was identified, some even saw broad consent as an obstacle for patients to make truly informed decisions. Shared by many was the fear of a loss of control over one’s personal health data:

“I think it's really difficult because (...) even if I sign some paper, so I agree to my data being used in this and that context and (...) I don't agree to any use beyond this context (...) from the outset or I am willing to an additional (....) expression of will, so to speak (…) that this will be extended but (...) in the end I have to say (...) that this is uncontrollable, right? The citizen XY cannot control this at all in the end, right?” (Member_2)

To members, broad consent does not offer sufficient control for patients. Representatives, in return, saw broad consent as articulated to the danger of de-anonymization, inadequate data protection or insufficient application of the EU General Data Protection Regulation (GDPR, German: “DSGVO”); the former often conceptualized as the result of the latter two. The GDPR is a is a European Union regulation on information privacy and was a standard point of reference for our interviewees.

A central issue for our interlocutors lay in the temporal dimension of broad consent. Excessively long periods of time covered by broad consent regulations were identified as problematic:

“but to promise to release it now for all eternity with an unknown destination - I'm simply actually still reluctant....” (Member_1)

This stance articulates two different dimensions: First, future data protection requirements are difficult to assess because of the unpredictability of both data protection technology and those technologies aimed at breaching protective systems.

“Because if I give it today, I can't be sure that the AI won't be able to decode it in five years' time. (…) But if I'm allowed to talk about it again in five years' time, then maybe I will, because today there may not be 100% certainty, but there is a relatively high degree of certainty that it won't happen.” (Representative_5)

Second, future requirements for knowledge (What kind of data is going to be important in the future?) cannot be adequately assessed from the presents’ point of view, nor can the development of the means to collect different kinds of data.

“it is of course also the case that certain framework conditions, whether it is from the outside, from society, from security (…) can simply change, the political situation can change, the inner attitude to certain things can simply change over time and (...) from my point of view it is difficult if you then (…) simply take a time window for five years and say yes, so here is a broad permission to use the data for this and there for that.” (Member_3)

For this reason, broad consent can only insufficiently account for, react to, and facilitate scientific progress and social change.

A second shared perspective is on questions of transparency and information on the one hand, and of concretization of a clearly defined research objective on the other. By and large, our interviewees identified precisely the crucial and defining feature of broad consent procedures as its major disadvantage: the lack of a clearly defined research purpose:

"[...] maybe it would be better for me to know what exactly is being researched so that (…) consent is obtained again and again for certain projects (...) so as far as I'm concerned, it can also be compiled so that you agree to one thing and not another is of course more work." (Member_4)

Whereas broad consent sets out to engage this lack of concretization in a productive way, precisely this is criticized as a major fault. A majority of our interviewees did not want to consent to secondary data use without knowledge about the purpose, trajectory, or outcome of the use of their data; this is especially true in relation to rare diseases:

“So I have to meet the patients halfway and say, look, you can donate to a project, what's your problem? But if you only ever want the big picture, it doesn't work. Especially not for patients with rare diseases. Because on top of that, patients who can be detected very quickly, even in pseudonymization, anonymization, have to be even more careful about what of their data is send out.” (Representative_5)

In most interviews, broad consent was equated with a lack of transparency; this built-in feature, however, was perceived as its main disadvantage. Interviewees criticized the purposeful omission of thorough and adequate information for the patients:

“But I think a lot of people will cringe when they are handed one of these broad consents at the front door. And I don't think it's good either, because if you open everything up, then you can't actually give patients any meaningful advice on what all this data is used for and how it is linked and who can and can't access it.” (Representative_1)

In return, those bits of information on general ways of secondary data usage provided in broad consent forms and procedures, some interviewees deemed as unintelligible. Therefore, some even rendered broad consent to be in violation of informational self-determination of the patients and thus to be in contradiction with the possibility of “consenting” sensu stricto:

“so we stand for or ultimately uphold the patient's informational self-determination. […] (...) In this context, this means that on the one hand, of course, as much information as possible must or should first be provided about what consent is to be given for in the sense of broad (…) consent, and what research is to be carried out with it. So that is always the prerequisite. Yes, you can only have effective consent if you have actually provided reliable information beforehand. That is one thing. And the other is that we advocate that in any case with these [broad] consents, which in this case is usually a patient or the citizen (...) should retain quite a bit of administrative sovereignty over it.” (Representative_3)

“because I don't know enough about what's happening, so I actually lack information to be able to make a good, informed decision.” (Member_1)

### Required measures: reflections towards other forms of consent

Interviewees proposed specific measures regarding consent regulations explicating that broad consent was not their preferred model. On the contrary, they demonstrated clear-cut preferences for alternative forms of consent, especially those balancing out the identified faults found in the temporal dimension, lack of information, and lack of concretization of the research objectives and outcomes.

The suggested measures are highly diverse, in parts singular, while in other parts we can identify preferences shared over a large amount of interviewees. One representative favored so-called “opt-out” solutions, sharing this wish with another representative and a member. Others, however, emphasized a desire for greater control of the data usage, while some suggested a choice of options for different consent procedures (and in regards different research purposes) in the hands of the patients:

“whether the consumer chooses a semi-granular solution and says okay, but I want to be able to control three, four, five buttons myself. And if you've asked me two or three times, then maybe I have so much confidence from using this whole, whatever, digital application and see that it makes life so much easier that I say okay, then I'll go for a more generous consensus, so to speak. And there are consumers who have their reasons for wanting to continue to fine-tune everything. That must all be possible, I think.” (Representative_6)

Far more interviewees opted for a clearer regulation in regards of the temporal horizon of the consent: they wished for a shorter time frame, with the option of updates or opting out – taking into account changes in regards data protection, novel scientific findings but also in terms of research purpose:

“Perhaps we can agree to make a broad consent, but then please renew it every five years. That's called revolving consent. (…) Interestingly, I have never heard researchers say that they always do it: There is no alternative to broad consent or informed consent. There is nothing in between. This revolving consent has several advantages, especially that if I as a patient am afraid of being re-identified, then of course I won't give my data.” (Representative_5)

Many demanded greater emphasis being put on issues of data protection. They regarded the security and reliability of the digital technologies involved in data storing and sharing as well as applications for consent and information as crucial for their willingness to consent to data usage. This is especially true in regards issues of anonymity:

“So if it is really certain that everything is anonymous, then yes.” (Member_5)

As a prerequisite, the risk of de-anonymization should be ruled out. Also compliance with the GDPR of both the consent procedure and the storing and sharing should be guaranteed.

Questions of “transparency” in regards both the practical aspects of consent procedures and in regards the research purpose were crucial to the stakeholders. A small group of members and about half of the representatives demanded a transparent consent procedure that provides an adequate time frame to make a reflected decision.

“But as long as we have this procedure that someone should actually be informed about what's happening, I don't think that's the right place and there's not enough time. And then to say to someone with, I don't know, some bad diagnosis, well, by the way, here's a stack of papers that you can sign quickly, it's good for you, I don't think that's right. So you should think about it and, if in doubt, be able to take it home with you and then make a decision as to whether you think it's right to give your data for the future or not.” (Representative_1)

To them, information is a key value in consent procedures; they wish for “good” and “useful” information provided to them by the institutions:

“So I don't think I would issue a complete carte blanche, but I think I am relatively willing to issue specific…. certain carte blanche if the information is good and useful (...) and to trust that science will use it sensibly.” (Member_1)

By and large, they called for “informed consent”. Demanding this, the stakeholders devalue broad consent procedures: the lack of a concrete research purpose is seen as a major fault. Accordingly, a majority of our interviewees shared the desire to be adequately informed about the specific use of their data and, thus, demanded the exposition of concrete research aims and goals as a prerequisite for consenting to secondary data usage:

“Yes, well, you only have effective consent if you've actually had a secure informed consent process beforehand.” (Representative_3)

Withal, this call is either formulated in more general terms or in specific suggestions as to the research purposes favored:

“Well, I would have a problem if my entire medical file was made available for research without (...) having a concrete (...) research approach in advance, so I just think that too much data would flow into research in that case without there being any benefit at all.” (Member_6)

## Discussion

In the interviews, we explicitly inquired whether a broad consent policy is supported. The interviewees often responded in rather general terms and related their statements to data sharing in general, independent of a specific consent procedure, especially where their answer was favorsome (see 3.1.). When being critical, however, they often referred specifically to broad consent procedures (see 3.2.). From our analysis we can show that stakeholders favor health data sharing and its secondary use for research and want to see this regulated by consent procedures. Yet, the favored procedures we identified are not necessarily comprehended by “broad consent”. Furthermore, the interviewees state specific understandings and wishes concerning the regulation of consenting to secondary date usage (see 3.3.).

On a preliminary view, and in line with the results of other German studies [22, 37–39], a significant number of interviewees initially express their favor to broad consent. However and in contrast to the aforementioned findings, our interlocuters often would then lay out specific ideas about appropriate procedures for regulating secondary data usage that diverge from classical renderings of broad consent (and, with that, from the findings of others). This indicates the value of in-depth, qualitative research and analysis engaging and accounting for the complex relations to and attitudes towards consent procedures of stakeholders [20, 49]. Such research can not only unbox the multiperspectivity of the subject matter but can furthermore account for it and make it matter in bio-ethical debates.

The analyzed material illustrates the intricacy of (individual) stakeholders’ perspectives on medical-ethical issues, showing that they are at least skeptical if not outright critical towards broad consent, all the while in favor of data sharing and secondary data usage. Thus, they affirm some aspects of broad consent while being critical towards others, thereby reflecting a much larger and overarching engagement with the topic. Our analysis makes apparent that broad consent is seen critical for exactly those features it was originally aimed at regulating [15]: unavailability of reliable information (“transparency”) as well as the long-term applicability (“temporal dimension”). For this reason, the stakeholders lay out specific regulations for secondary data usage and streamlined consent procedures that are not compatible with broad consent sensu stricto: Their proposals involve a re-thinking of the questions of transparency and information as well as the desired time-span of consent procedures. With this, our interlocutors’ perspectives resonate with alternative forms of consent discussed in the literature [7, 24]. Specific suggestions of our interlocutors are found in “dynamic consent” models [11], that features a built-in-possibility of interaction and communication, albeit covers an extended duration,or in “layered consent”, that shares the “unspecificity” of broad consent but counters this by adding additional layers providing more detailed information upon request [6]. Some favor built-in “opt-out” features informing participants about their right to withdraw the use of their personal data [12]. Others demand a dimension of choice and control and, thus, opt for meta- or granular-consent procedures giving more options to choose from (i.e. what kind of consent they want to give, such as informed, opt-out, layered- or broad consent) and an individual “consent portfolio” can be fashioned [33].

That means in conclusion, while taking a generally positive attitude towards data sharing and secondary data use and supporting the cause of freedom of research and the improvement of treatment methods, the stakeholders demand transparency and a level of control and participation. They frame this demand ethically as “reciprocity” for making medical data available. In doing so, however, they conceptualize “informed consent”, with its requirement of decisions being based on sound information, as the gold standard of consent procedures and reject “broad consent” as their preferred procedure to regulate secondary data usage. While participants referenced various alternatives—such as dynamic consent, periodic re-consent, and transparent reporting—no single governance structure was consistently emphasized across interviews. This diversity of views underscores the complexity of consent governance and the need for flexible, context-sensitive approaches. For further research this suggests the need for both studies on ethically robust and practically feasible models and studies that further explore regulatory frameworks that can accommodate these varied expectation.

### Not “informants”, but “experts” (reflexivity of the interviewees)

Our interlocutors – both representatives and members – appear not simply as lay- or patient-informants but as simultaneously “affected” and “experts” (cf. [35, 36]). Our analysis shows a high degree of reflexivity in regards the intricate issues of data sovereignty, data security, and secondary date usage, but also of patient autonomy, responsibility, and accountability of medical research. Our interviewees are therewith according to us reflective experts navigating the complex field of general, but highly relevant bio-ethical issues. They demonstrate a great level of knowledge (on secondary data usage, on health policies, on clinical research etc.) and reflection (e.g., on the limitation of consent procedures, on technical conditions, or on ethical dilemmas). The problem we identify is, therefore, less to be found in the necessity of better information on different consent procedures, as suggested in the literature [28, 34]. From our perspective, the problem in the consent regulation of secondary data usage is a lack of a) in-depth qualitative and descriptive analysis of stakeholders’ perspectives in their double position as experts and affected persons and b) a lack of participation and active engagement of these stakeholders as a potential result of said analysis.

### Towards a recommendation: is a dynamic broad consent procedure a possible way to go?

Currently, the bio-ethical debate on consent forms focuses on what form of consent adequately respects patient autonomy [7, 24]. Considering the three main critical aspects of transparency, control, and time span, we suggest an alternative route for implementing (broad) consent procedures, shifting the focus away from the form of consent towards how it is embedded within a concrete consent praxis.

Since transparency regarding research aims cannot (always) be given at the time of data acquirement, consent procedures should be situated within a praxis of ongoing, transparent communication. To this end, we propose a two-way communication system (e.g., a digital app) through which patients who have given their (broad) consent are regularly informed about the studies, both finished and planned, that use their data. Based on this information, patients can retract their consent—either entirely or selectively for specific studies.

We refer to this model as a *dynamic broad consent* procedure. While it shares features with the established concept of *dynamic consent* (see [11]), we argue that it differs in scope and emphasis. Dynamic consent typically presupposes study-specific consent updates and continuous engagement, which may be impractical in large-scale data infrastructures. In contrast, dynamic broad consent retains the general nature of broad consent but enhances it with mechanisms for transparency and selective withdrawal, thereby offering a more feasible and ethically responsive model for long-term secondary data use. It is crucial to provide patients with both the transparency and control over the health data they make available for (secondary) use over a longer time horizon.

### Suggestions for further research

As discussed above, our sample can be viewed as “experts of experience “, as they all have thorough experience with the digital health care system such as participating in the development of a patient registry. While this provides valuable insights into ethically informed perspectives on broad consent, it also offers suggestions for further research. The inclusion of chronically-ill patients and their representatives may have created a positive bias toward secondary data use, as these individuals may perceive research participation as part of their advocacy [2]. Furthermore, the sample does not reflect the full diversity of patient populations—such as those with limited health literacy, no prior research exposure, or restricted access to digital technologies, particularly in rural or underserved settings. Future research could include also such patient populations to extend on our insights and provide further ground to develop inclusive and context-sensitive models of broad consent that can account for varying degrees of education, technological access, and trust in research institutions.

## Conclusions

The interviewees consider the provision of data for secondary use to be important for the improvement of treatment methods, freedom of research, and ethical considerations such as solidarity. However, these values are linked to conditions in the stakeholders' considerations and thus appear less absolute and universal than conditional and situated. In the scientific literature on the matter, the problem seems to be elevated in abstraction [8, 21, 48]. The issue raised in a number of contributions, is, to the authors, located in the question of how to appropriately inform patients about the different procedures of consent in general, and the specific application of “broad consent” and its concrete implications. The problem of appropriate information is detached from questions of information about research objectives and the specific use of the research data in question. Conversely, “appropriate information” is conceptualized in terms of an improvement of “consent literacy” on the side of the patients: How can patients be enabled to consent to and make informed decisions about different forms of consent? Patients, in conclusion, are to be enabled to “consent to consent” [34]. However, our empirical study shows that the problem is not a problem of “information” in a narrow sense, but one of lacking two-way-communication with relevant stakeholders. On the one hand, this discrepancy points to an increased need for theoretical-conceptual research against the background of our findings. On the other hand, these findings not only indicate the need for empirical-ethical research with stakeholders. It simultaneously underscores the necessity of research on how to adequately include stakeholders, such as patient organizations and their representatives, into the development of consent procedures that are aimed to the provision of data for secondary use.

## Supplementary Information

Additional file 1. COREQ checklist

Additional file 2. Overview of interview questions

Additional file 3. Coding guides

Additional file 4. Quotes in the German original

## Data Availability

The datasets used and/or analysed during the current study are available from the corresponding author on reasonable request.

## Abbreviations

PO: Patient organizations
PANDORA: Patient-centered digitalization: an ethical analysis of the role of patient organizations as actors in the context of digitalization in health-related research and care
GDPR: General data protection regulation
