# Supplementary material for: Critical consideration towards broad consent by patient experts: results of a semi-structured interview study on the secondary use of medical data
Source: BMC Med Ethics. 2025 Nov 18;26:163. doi: 10.1186/s12910-025-01326-x (PMC12629026; doi:10.1186/s12910-025-01326-x)
Supplement: Supplementary file 2 — Additional file 2. Overview of interview questions [file 12910_2025_1326_MOESM2_ESM.pdf]

UNIVERSITÄTSMEDIZIN GÖTTINGEN UMG

HAW HAMBURG

Hochschule für Angewandte Wissenschaften Hamburg, 21033 Hamburg
Departement Gesundheitswissenschaften, Ulmenliet 20, 21033 Hamburg

Project lead: Prof.'in Dr. Sabine Wöhlke

Universitätsmedizin Göttingen, 37099 Göttingen
Abteilung Ethik und Geschichte der Medizin, Humboldtallee 36, 37073 Göttingen

Project lead: Prof.'in Dr. Silke Schicktanz

Project employee: Dr. Henk J. van Gils-Schmidt, B.Sc.

Ulmenliet 20, 37073 Hamburg **Adress**
040 428 75-6262 **Telephone**
Henk.vanGils@HAW-Hamburg.de **E-Mail**

19. September 2022 **Date**

Interview Guide

Digitization projects in medical research and care

Patient-oriented digitization: An ethical analysis of the role of patient organizations as actors in the context of digitization in health-related research and care (PANDORA)

Italics = follow-up questions that are only asked if the interviewee's previous answer is very brief or not very informative.

Questions marked in blue are only for people who have actively decided in favor of data donation.
Questions marked in yellow are only for people who have actively decided against donating data.

Before the interview:

- Greeting, interviewer introduces him/herself (very briefly)
- Reassurance about the time frame available to the interviewee for the interview
- Repeat information about the recording of the interview and the confidential handling of information
- (if not yet received) request to send the signed declaration of consent
- Repeat explanation of the background to the study
- Request to complete questionnaire (regarding demographic data). Please note that the answers will remain anonymous and that the interviewer will not see the questionnaire before the interview.
- Explain: Researchers' interest in personal views, attitudes and evaluations; request for personal assessment (as an experienced person). No right or wrong

A) Introduction: personal background

1) Would you like to briefly introduce yourself?
    a. *Name, place of residence, occupation*

B) Opportunities and risks of digital applications in medicine (general)

- Explain: To begin with, a few general questions regarding digital applications in the medical field.

2) What does "digitalization" in medical research and care mean to you? What specific examples do you know or have you dealt with in the past?
    a. *If applicable, ask: Which e-health products would you support?*
    b. *If applicable, ask: What opportunities and risks do you see in the use of such digital products?*
    c. *If applicable, ask: Do you perceive a difference between the research and care context?*
    d. *If necessary, explain: E-health means "health based on electronic data processing" and is a generic term for digitalization in the medical field. E-health refers to the category of medical applications and measures that focus on the digital support or digital shift of medical diagnostics, treatment and care. E-health products can be offered by private companies, associations and organizations as well as public healthcare providers.*

3) What do you understand by health literacy? And what level of competence do you consider necessary for you or other people to be able to participate in digital health projects and technologies?
    a. If necessary, explain: Health literacy can be understood as the knowledge, motivation and skills of people to find, understand, assess and apply relevant health information in everyday life.

4) The majority of communication is now carried out using cell phones, tablets, etc. In addition to health literacy, digital literacy is therefore becoming increasingly important. How would you describe digital literacy? What level of competence do you consider necessary for you or other people to be able to participate in digital health projects and technologies?
    a. If necessary, explain: Digital literacy is the knowledge, motivation and skills of people to participate in everyday digital life and to handle, learn, work with and critically evaluate digital applications.

5) In your opinion, what responsibility do participating organizations have in the implementation of digital health projects? Those involved can be, for example, PO/SHO, private companies, science or politics. Would you assign different areas to the different stakeholders? What is the difference, if any?

C) Opportunities and risks of digital electronic patient registers

- Explain: I would like to ask a few questions on the subject of patient registers.

6) How do you rate the initiative of your PO/SHO to open a patient registry?
   a. *Ask members of Mukoviszidoze e. V: Have you received information and materials from your doctor to inform you about participating in the patient register? Is this information available on the PO's website for you to read?*

   b. *If applicable, ask: main reasons for deciding for / against*
   c. *If applicable, ask: Assessment of health benefits*
   d. *If applicable, ask: Benefits for society or sense of community for the community of those affected*
   e. *If applicable, ask: Possibility to control/determine your data and its use or the role of data security*

7) To what extent do patient registries present opportunities and challenges as to why people do or do not consent to data use?

8) What information and materials have you received from your doctor to inform you about participating in the patient register?
   a. *If applicable, ask members of Mukoviszidose e.V.: Mukoviszidose e.V. has made the documents for the register available on its homepage. Were you given these documents by the doctor treating you?*
   b. *If applicable, ask: are there any further information you would have liked to receive?*

9) We have already talked about the responsibility that a PO/SHO should take in the implementation of e-health initiatives.
   What specific measures do you consider important in the implementation of a registry?
   a. *If applicable, ask: is this about data protection, about the use of the data or also about what is being researched with it?*
   b. *Possible actors that can be explicitly mentioned (see question 5): SHO/PO, private companies, academia, politics*

10) Consent to donate personal data to a patient registry can be given in different ways. Either the data is used for a specific study or for a broad range of future research projects, the content of which cannot yet be foreseen at the time of consent. What is your opinion on this broad consent?
    a. What disadvantages/advantages do you see in it?
    b. *If applicable, ask: Are there any measures that can strengthen your sense of security with regard to the use of your personal (sensitive) data in the context of broad consent?*

11) Are there any experiences and opinions that you would like to address that have not yet been addressed?

Exit from the interview:

- Invitation: We would be delighted if you would continue to participate in the PANDORA project! Collaboration is desired in helping to shape and find topics for (video)podcasts; the project team will also be happy to receive suggestions at a later date.

- Farewell

- Reminder: Please note that you can contact us at any time with questions etc.

- Reminder: Please note that the recordings will be treated in accordance with the data protection guidelines

UNIVERSITÄTSMEDIZIN GÖTTINGEN UMG   HAW HAMBURG

Questionnaire on demographic data

Please enter your gender:

☐ Male ☐ Female ☐ Diverse

Please enter your age:

… Years

Which patient organization(s) are you a member of?

………………………………..

What is your relation to the patient organisation?

☐ Patient myself

☐ Family of a patient

☐ Caretaker of a patient

☐ Acquiantance of a patient

What is your highest school degree?

☐ No qualification

☐ Secondary school certificate (Hauptschulabschluss)

☐ Secondary school certificate (Realschulabschluss)

☐ University entrance qualification/ A-levels

☐ Other

What is your highest vocational training?

☐ No training

☐ Recognized training

☐ Master craftsman examination

☐ University degree (Bachelor/Master/Diploma)

☐ Doctorate

☐ Other

Interview guide_Representatives of Patient Organisations (English translation of original German)

UNIVERSITÄTSMEDIZIN GÖTTINGEN UMG

HAW HAMBURG

Hochschule für Angewandte Wissenschaften Hamburg, 21033 Hamburg
Departement Gesundheitswissenschaften, Ulmenliet 20, 21033 Hamburg

Project lead: Prof.'in Dr. Sabine Wöhlke

Universitätsmedizin Göttingen, 37099 Göttingen
Abteilung Ethik und Geschichte der Medizin, Humboldtallee 36, 37073 Göttingen

Project lead: Prof.'in Dr. Silke Schicktanz

Project employee: Dr. Henk J. van Gils-Schmidt, B.Sc.

Ulmenliet 20, 37073 Hamburg **Adress**
040 428 75-6262 **Telephone**
Henk.vanGils@HAW-Hamburg.de **E-Mail**

12. Oktober 2022 **Date**

Interview Guide

Digitization projects in medical research and care

Patient-oriented digitization: An ethical analysis of the role of patient organizations as actors in the context of digitization in health-related research and care (PANDORA)

Italics = follow-up questions that are only asked if the interviewee's previous answer is very brief or not very informative.

[illegible] Interview:

- [illegible]
- [illegible]
- [illegible]
- [illegible]
- [illegible]
- [illegible]
- [illegible]

A) Introduction: Personal background/expertise with digitalization

1) What is your professional background? What is your role in your organisation?

2) What experience/expertise do you or your organization have with digitalization in the field of medical research and care?
    a. *If applicable, ask: What digitization projects are currently being discussed in your organization?*
        i. *Big data/personalized medicine; m-health; collaboration with external actors; who initiated the discussion (leadership; members; external actor)*

B) Ethical opportunities and risks of digitalization in the field of medicine

3) What opportunities and risks does your organization expect from the use of digital technologies in the medical field?
    a. *If applicable, ask: specific ethical opportunities/risks?*
    b. *If applicable, ask: Despite perceived risks, would digitization projects be required because of the benefits?*
    c. *If applicable, ask: Difference between collecting data in the context of big data and patient registries & other types of e-health applications, such as telehealth or supporting apps for e.g. determining vital signs*
    d. *If applicable, ask: specific benefits or advantages for the group of people affected that your patient organization represents*
    e. *If applicable, ask: Are there any specific obstacles that make it difficult for the patients represented by your PO/SHO to participate in digitalization?*

4) What ethical issues are important to you in the use of digital technologies in the medical and/or research field?
    a. *If applicable, ask: What are your previous experiences and/or activities in connection with ethical aspects of digitalization projects?*
    b. *If applicable, ask: Has trust been an issue discussed here for your organization?*

5) In your organization's opinion, is the current regulation of the use of digital technologies and the use of personal data in the medical field sufficient to protect data subjects? What could or should be improved?
    a. *If applicable, ask: Who should be responsible for regulation? What role do patient organizations have in this?*
    b. *If applicable, ask: Who should (not) have access to personal data*
    c. *If applicable, ask: How should patients' control over their personal data be organized?*
        i. *If applicable, ask: Relationship between data sovereignty and solidarity*

6) How would PO change as a result of digitalization? Here we are interested in the services offered as well as the procedures and processes within the organization.

C) Digital applications and the people affected

7) Have specific complaints or problems been raised by your members in previous digitization projects in which your organization has been involved? If so, could you explain them?
    a. *If applicable, ask: How did your organization deal with such complaints or problems? Have they been taken into account and, if so, how?*

8) Have you involved members of your organization in digitalization processes or designed them together?
    a. Could you give specific examples?
    b. How important is it for you to shape digitalization together with your members? Why?

9) What measures have been taken to strengthen members' trust in digitalization projects?
    a. What potential problems could burden/endanger the trust of members?

10) Patients are increasingly using health-related digital apps themselves or participating in e-health and digitalization initiatives such as digital patient registers, telehealth and monitoring via apps. What changes do you see resulting from this for the role of patients?
    a. If applicable, ask: What health literacy skills do patients need to be able to make independent decisions about their use and participation?
    if asked, explain: Health literacy: the knowledge, motivation and skills of people to find, understand, assess and apply relevant health information in everyday life.
    b. If applicable, ask: What information do patients need to be able to make independent decisions and ensure responsible handling of data?
    c. If applicable, ask: In your opinion, what are the desirable and problematic changes here?

11) In which areas could patients be more actively involved in research/digitalization projects? Where do you see the greatest challenges in this respect?

a. *If applicable, ask: Difference between collecting data in the context of big data and patient registries & other types of e-health applications, such as telehealth or supporting apps for e.g. determining vital signs.*
b. Follow-up question: are there limits to possible and/or desired participation?

12) In the context of research initiatives that analyze large amounts of data (so-called big data), there are efforts to replace the individual informed consent of participants with so-called broad consent. What is your organization's position on such a proposal?
    a. What ethical risks or problems do you see with such a broad, general declaration of consent?
    b. *If necessary, explain: Broad consent means that patients are asked to give their consent to a wide range of future research projects, the content of which cannot yet be foreseen at the time of consent.*

13) Are there any experiences and opinions that you would like to address that have not yet been addressed?

Exit from the interview:

- Invitation: We would be delighted if you would continue to participate in the PANDORA project! Collaboration is desired in helping to shape and find topics for (video)podcasts; the project team will also be happy to receive suggestions at a later date

- Farewell

- Reminder: Please note that you can contact us at any time with questions etc.

- Reminder: Please note that the recordings will be treated in accordance with the data protection guidelines

UNIVERSITÄTSMEDIZIN GÖTTINGEN UMG

HAW HAMBURG

Questionnaire on demographic data

Please enter your gender.

☐ Male ☐ Female ☐ Diverse

Please enter your age:

… Years

Which patient organization(s) are you a representative of?

………………………………..

What is your role in the organization?

………………………………..

What is your highest school degree?

☐ No qualification

☐ Secondary school certificate (Hauptschulabschluss)

☐ Secondary school certificate (Realschulabschluss)

☐ University entrance qualification/ A-levels

☐ Other

What is your highest vocational training?

☐ No training

☐ Recognized training

☐ Master craftsman examination

☐ University degree (Bachelor/Master/Diploma)

☐ Doctorate

☐ Other
