# Supplementary material for: Critical consideration towards broad consent by patient experts: results of a semi-structured interview study on the secondary use of medical data
Source: BMC Med Ethics. 2025 Nov 18;26:163. doi: 10.1186/s12910-025-01326-x (PMC12629026; doi:10.1186/s12910-025-01326-x)
Supplement: Supplementary file 3 — Additional file 3. Coding guides [file 12910_2025_1326_MOESM3_ESM.pdf]

This is an English translation of the original German coding guide

Coding manual for members of PO fort he interview study *“Digitization projects in medical research and care“* (as at 24.03.2023)

Dr. Henk Jasper van Gils-Schmidt, B. Sc.
Department of Health Sciences, Faculty of Life Sciences
HAW Hamburg
Ulmenliet 20
21033 Hamburg

<u>Definitions</u>

Digitization: *Digitization refers to the conversion of analogue data and information into digital formats. In a broader sense, digitization also describes the transformation of social practices that have taken place in analogue or in person into forms in which these practices are mediated by digital applications.*

Patient registry: *A patient registry is usually a (digital, electronic) database system in which the personal data of patients with a specific disease is recorded. In such a registry, not only disease characteristics are recorded, but also (drug) treatment methods tailored to them.*

Patient organization: *Patient organizations represent the interests of people with disabilities or illnesses and are committed to improving the care of those affected and strengthening the skills of those affected in dealing with their illness or disability. Many patient organizations are also politically active, engage in national and international networking, support medical research and initiate their own research projects.*
Patient organizations are usually associations of patients and/or their relatives. They do not primarily pursue economic goals and are usually non-profit registered associations.

E-health: *E-health (short for electronic health) means "health based on electronic data processing" and is a generic term for digitalization in the medical field. E-health refers to the category of medical applications and measures that focus on the digital support or digital relocation of medical diagnostics, treatment and care. E-health products can be offered by private companies, associations and organizations as well as public healthcare providers.*
Examples of such products include electronic patient files, digital patient registries, telemedicine for communication between doctors and patients, online pharmacies, prescription apps, bloodless glucose measurement for diabetics and emergency call buttons for senior citizens.

M-health: *M-Health (short for mobile health) refers to a subset of e-health activities and measures in healthcare that are offered on mobile, wireless devices such as smartphones or tablets. The areas of application include determining vital signs such as blood sugar or body temperature (see remote monitoring) or motivational applications that remind people to take their medication, for example (see self-management of illness).*
Examples of M-Health products are: Corona-Warn App (the official tracking app by the German federal government), applications for monitoring vital functions (e.g. blood pressure monitors, blood glucose meters), wearables (e.g. fitness wristbands).

Health literacy: *Health literacy encompasses the knowledge, motivation and ability of people to find, understand, assess and apply relevant health information in everyday life, for example to make informed health-related decisions. Health literacy also refers to people's ability to find their way around the healthcare system, identify suitable services and facilities and successfully claim their own benefits.*

Digital literacy: *encompasses the knowledge, motivation and skills of people to participate in everyday digital life and to use, learn or work with and critically evaluate digital applications.*

Broad consent: *Broad consent is a type of consent form in which patients agree that their data may be used in future research projects without the need for renewed consent. The content and scope of these future research projects are not yet known at the time of consent. The broad consent should make it possible to reuse existing data for new research purposes without having to go through a lot of organizational effort. Broad consent should also make it easier for the people who give their consent to contribute to research and prevent them from being overburdened by constant new requests for consent. The broad consent model is therefore intended to relieve the burden on researchers and patients and save costs by reducing the administrative burden. One criticism of the broad consent model is that it can jeopardize the autonomy of the patients whose data is collected and processed.*

Questions

- Which digital services for medical research and/or care are known to members of patient organizations? Is there a perceived difference between offers in the field of medical research and in the field of medical care?
- How do members rate digital services? Is the assessment different in the context of research and healthcare?
- What is the assessment of which aspects of health literacy and digital competence are necessary in order to be able to use digital services effectively and make self-determined decisions regarding such services?
- What areas of responsibility are perceived in the development and use of digital services? And who are the stakeholders who must fulfill this responsibility?

- What attitudes do members have towards the digital patient registry of their own patient organization?
- What reasons for and against participation in the registry are made explicit?
- What measures are the responsibility of the patient organization that (co-)initiated the registry?

- How do PO members evaluate broad consent in the context of research studies?

	Code	Description/meaning, incl. application of the code	Not to be confused with	Anchor example
Digitalization – generel				
1.	Knowledge_Dig Tech	Naming or describing digital technologies in the context of medical research or care. This means that the interviewee names or describes a digital technology, such as:<br>1. PR (Patient registry)<br>2. AI (Artifical Intelligence-applications)<br>3. BG (Big Data)<br>4. TM (Telemedicine)<br>5. eRecept<br>6. ePA (electronic patient file)<br>7. DiGA (digital health applications of which the use are paid for by health insurance companies)<br>8. M-Health (Health apps that are not digital health applications)<br><br>*Note: This code can be subdivided into subcodes according to specific technologies later in the process, based on the reading of the transcripts (= inductive). This would make it possible to analyze the answers by technology in later codes (e.g. degree of health literacy, code 6).*		*Examples for naming technologies:*<br>„So I'm thinking of registries, we'll get to that later , […]"<br><br>„[…] So my electronic patient file is still too complicated for me. [I: Okay.] I also don't use DiGA because I simply don't have the opportunity […]"<br><br>*Example for describing technologies (telemedicine):*<br>„I've heard about it yes (...) specifically (...) yes, I think there are video consultations that GPs are already offering (...) I think in psychotherapy this is or is becoming widespread that telemedicine consultations are being offered there too […]"
2.	Attitude_Dig	It describes attitudes specifically towards digitalization in medical research and/or care or the importance of this digitalization. This means that this code has an evaluative moment, which can be both pro and contra.		

	Code	Description/meaning, incl. application of the code	Not to be confused with	Anchor example
		It is not used when *only* reporting on personal experiences or discussing digital products.<br><br>**Only the subcodes of this code are applied.**<br>*(Note: This main code can be combined with the subcodes in the analysis.)*<br><br>***Note**: This code can be subdivided into further subcodes later in the process, based on the reading of the transcripts (= inductive), if necessary according to the difference between "research" and "care".*<br><br>***Note:** When coding or analyzing, attention should be paid to whether a clear distinction can be made between concerns (e.g. value of privacy) and motives (e.g. solidarity as motivation). This should be coded additionally if necessary.*		
2.1	Attitude_Dig_Pro	This subcode is used if the described attitudes towards digitalization in the medical field are generally positive (even if it is unclear whether research or care is specifically meant).	Exp_Dig_self AND Attitude_Dig_Con	„Well, I don't know to what extent you now (...) include treatment (...) via telemedicine. So for me it seems to be a very good option and it means that patients and doctors can be connected over long distances."<br><br>„[…] So especially with the electronic patient file, I can actually decide for myself what goes on it or not and what I approve or not (...) and (5 sec.) so for me at the moment I only see opportunities there […]"<br><br>„[…] I also think the DiGAs are a very good thing in the long run, these digital applications with which you can control your illness yourself, they also bring new requirements, but I think in principle it's a very good thing […]"

	Code	Description/meaning, incl. application of the code	Not to be confused with	Anchor example
2.2	Attitude_Dig_Con	This subcode is used if the described attitudes towards digitization are negative (even if it is unclear whether research or care is meant specifically).	Exp_Dig_self AND Attitude_Dig_Pro	„At the moment I'm not convinced of the benefits for me personally, because I still like to collect my data on paper, which is enough for me at the moment. I can imagine that if it is well developed that you really have a benefit because you carry the results around with you everywhere, but I have to be honest and say that I am not at all sure whether I ALWAYS want ALL my data to be accessible, i.e. viewable, through it.“<br><br>„[…] We've also thought about whether the development of telemedicine might also harbor the danger that hospitals might use it as an excuse to cut back on real care, where a doctor is also physically available (laughs) and takes time for the patient in order to save costs, because they say yes, we have telemedicine, so of course you always have to be careful what effect a change has and what it means for patient care […]“<br><br>„So one (...) I know that the patient representatives are also concerned about the (PO[2]) that the patient representatives are generally very worried about data leaks, about data falling into the wrong hands, being used for manipulation or to exclude entire groups of patients, for example in the insurance sector, and of course I also see these risks. (...) So you keep hearing about data leaks, that the KBV (laughs) suddenly has a huge data leak and so on and that this data falls into the wrong hands and that is of course a risk.“
3.	Exp_Dig_self	Personal experiences that the interviewee has already had with digital applications or their use. Specific examples of the use of digital services are meant.	Einstellung_Dig AND Exp_Dig_other	„[…] For me personally, the new possibilities via apps and smartwatches are also very relevant, so that you can record your own movement profiles, for example, so when I run, I record my runs and also my heart rate and can monitor myself and also recognize my progress and then of course share it on social media

	Code	Description/meaning, incl. application of the code	Not to be confused with	Anchor example
		This code is used when personal experiences are discussed. If an evaluative moment is included here, then a subcode of “Attitude_Dig” must also be coded.		(laughs), so tell others what you have done, which helps to motivate each other, so that is also largely a part of medicine for me, because I run to train my lungs (. ...). ...) so there are links to the training sessions.“<br><br>*Example of double coding with Attitude_Dig (Attitude_Dig is highlighted in yellow to highlight it):*<br>„That is (...) yes, I would like to use it, but I have the feeling from everything I've seen so far that it's just words, but it doesn't even begin to work. [I: Okay.] (...) So I was actually planning to use the electronic patient file, or I've also registered (...) to use the electronic patient file, as I said at the moment I've saved diagnoses or reports of findings when I get them on my smartphone or tablet and I imagined that you could also save them on the chip of the health insurance card. But from what I've read now, this doesn't work at all with my health insurance company.“
4.	Exp_Dig_other	Experiences that the interviewee has had indirectly via relatives with digital applications or their use. This refers to specific examples of the use of digital services.<br><br>If an evaluative moment is included here, then a subcode of “Attitude_Dig” must also be coded.	Exp_Dig_self	„Yes, well, most people (clears throat) already know a bit about computers and the Internet (...) that's just (...) hm [I: Yes.] (...) so hm in my private environment, again the two extremes I have to do with it professionally (...) and try out a lot more and look a lot more and also know what she has to pay a bit of attention to […]“<br><br>*Example of double coding with Attitude_Dig (Attitude_Dig is highlighted in yellow to highlight it):*<br>„[…] so at least the people I know are all older, of course, so there are a lot of older people, and they have much less contact with digital processes than I do now, they are just very, very insecure about it.“
Digital literacy Kompetenz & health literacy				
5	hL_Aspects	They report on their own understanding of health literacy and/or the aspects that make up health	dL_Aspects AND	„Well, I would describe health literacy on the part of the patient [I: Yes] as being able to adequately answer or solve the (...) health-

	Code	Description/meaning, incl. application of the code	Not to be confused with	Anchor example
		literacy. These aspects can either be named directly or they can be illustrated using everyday examples. Examples include the person describing certain health knowledge, motivations or skills of patients.<br><br>The understanding of health literacy does not have to be related to digitalization in the medical field or e-health services, but this is possible.	hL_Preconditi ons	related questions that I have. That means that I am able to know where to ask for information, where to get valid information, where to find information, how to integrate it, where I want to have information and where I don't want to have information. (...) So that I can make conscious decisions about my health […]"<br><br>„Well, I think that's just a personal attitude. (...) Well, I'm someone who always wants to know everything, so I ask things that don't necessarily have to do with the findings and then I think of something like how does this work and what's there and I always want to know everything exactly, I always want to know when I'm operated on how exactly the operation works. (laughs) (...) Preferably with video and stuff like that […] "
6	hL_Preconditio ns	The interviewee names or describes the general prerequisites that patients need in order to be able to participate in health projects or use health applications.<br><br>This code describes either the prerequisites or conditions necessary for good participation in the healthcare system, or a level or degree of health literacy.<br><br>***Exception***: *If the prerequisites for health literacy or the level of health literacy are addressed at the same time as and digital literacy, both codes should be assigned.*	hL_Aspects AND dL_Preconditi ons<br><br>[Exception: see description]	„[…] depending on the severity of the disease, so (K[1]) is fatal per se and the therapy measures are incredibly time-consuming, so I used to have to do up to four hours of respiratory therapy a day and the more I understand about it and the more I optimize it, the more effective it is and the more I can limit this time expenditure and only then is it possible to work on the side and (laughs) do other things, so from that point of view [I: Yes] I would say always achieve the highest possible level of the best possible competence […]
7	dL_Aspects	They report on their own understanding of digital competence and/or the aspects of which it consists. These aspects can either be named directly or they can be illustrated using everyday examples. Examples	hL_Aspects AND dL_Preconditi ons	„So the ability to understand how I charge something, for example, how I identify and authenticate myself on the network then the gadget or that, to get the applications to work then to understand

	Code	Description/meaning, incl. application of the code	Not to be confused with	Anchor example
		include the person describing specific knowledge about digital technologies, motivations or skills of patients.<br><br>The understanding of digital competence does not have to be related to digitalization in the medical field or e-health services, but this is possible.		the application what it has to do, so possibly to understand the graphics that are shown to me […].“
8	dL_Preconditions	The interviewee names or describes the general prerequisites in terms of technological means or digital skills that patients need to be able to participate in digital health projects or use digital applications.<br><br>This code describes either the prerequisites or conditions necessary for participation in digital technologies in the healthcare system, or a level or degree of digital competence required to participate in digital health projects.<br>To assign this code, there must be a connection to e-health and digital health projects.<br><br>***Exception***: *If the prerequisites for digital literacy or the level of digital literacy are addressed at the same time as health literacy, both codes should be assigned.*	dL_Aspects AND hL_Preconditions<br><br>[Exception: see description]	„Well, you just need a basic understanding of IT processes, I would say.“<br><br>„[…] Of course, a bit of basic digital understanding is necessary […]“<br><br>*Example of double-coding:*<br>„So then he must at least understand how it works, using the example of the sugar sensor, he must be able to register the sensor on the iPhone or on the phone, he must be able to recognize when an error occurs somehow and the values that are displayed there may not be correct, so he must know what is normal, how are the values to be assessed, what are the consequences if I get a certain value displayed and (...). ...) so digital competence is also the ability to send the evaluations to the doctor to be able to log in and log out Login what do I know all these technical things he has to master so far that he can use these applications with a good feeling.“
Responsibility				
9.1-6	Responsibility_[Stakeholder]	Actors for whom the interviewee states that they have a responsibility or competence to take measures to protect patients who use digital services (e.g. data protection regulations) or to support them (promotion of skills in the use of digital technologies).		*Note: Here below is coded with colors to show how subcodes can follow each other. In MAXQDA, however, this text passage must be coded separately according to the subcodes. Example for 9.1, 9.3, 9.5 and 9.4:*<br>„[…] So with the EPA (laughts) it‘s principly (clears throat) the politicians are responsible for initiating the whole thing in the first

	Code	Description/meaning, incl. application of the code	Not to be confused with	Anchor example
		If the areas or aspects of responsibility identified for the individual actors are mentioned, these are coded directly as well.<br><br>A distinction must be made here between:<br>1. Politics<br>2. PO (patient organizations)<br>3. HS (actors from the healthcare system, such as hospitals, doctors, etc.)<br>4. manufacturers of digital products<br>5. Users (patients, etc.)<br>6. Miscellaneous		place, (...) then of course I must honestly say I don't know exactly what this committee is called that is supposed to develop and promote the EPA, then the responsibility lies with those involved to carry out the project in an appropriate form, then it would be up to the patient to decide yes or no, so I want to download the EPA, do I want to use it or not, at least for a certain period of time. , und then it is up to the doctors to deal with this EPO in a correct or appropriate way.“<br><br>*Note: Here below is coded with colors to show how subcodes can follow each other. In MAXQDA, however, this text passage must be coded separately according to the subcodes. Example for Textpassage for 9.1, 9.3 and 9.5:*<br>„ So, (...) since I am convinced that patient registries can advance the progress of therapy and are therefore important for the patients and of course also offer the individual patient a service that is useful for coping with the disease, I think that the healthcare system as a whole should have the task of financing such patient registries and that is the responsibility for financing, so to speak (laughs) and that is ultimately the task of politics to set guidelines there (…) But nevertheless, they are not the only ones who bear responsibility, so of course the doctors and practitioners also bear responsibility for entering data correctly, for being committed and taking the time to generate the data in the first place, for discussing it with the patient and using the results of the registry We have very different experiences here, so there are outpatient clinics here that use every feature of the registry and make it immediately accessible to patients, and for others it runs on the side and is handled rather lovelessly[I: Okay.] so (…) and the responsibility of the patient to provide their data, otherwise it won't work.“
The digital Patientenregistry				

	Code	Description/meaning, incl. application of the code	Not to be confused with	Anchor example
10	Attitude_dPR_self<br>[**Use only the subcobes**]	Attitudes and experiences that the interviewee has about the digital patient registry of their own patient organization are described. These attitudes also include the advantages and disadvantages perceived by the interviewee.<br><br>It is not used if *only* their own experiences regarding the registry are reported.<br><br>**Only the subcodes of this code are applied.**<br>*(**Note:** This main code can be summarized in the analysis with the subcodes.)*<br><br>***Note:** In the coding or analysis, attention should be paid to whether a clear distinction can be made between concerns and motives. This should be coded additionally if necessary.*		
10.1	Attitude_dPR_self_Pro	This subcode is used if the described attitudes and experiences that the person has themselves are positive towards the digital patient registry or if benefits of the registry are mentioned that the person themselves perceives.	Attitude_dPR_other	„[...] but it was clear to me from the beginning that the registry is a very important institution or a very important means of (...) documenting and evaluating verified data from patients and thus describing the course of the disease. [I: Yes.] (...) And to describe the effectiveness of measures.“
10.2	Attitude_dPR_self_Con	This subcode is used if the described attitudes and experiences that the person themselves have are negative towards the digital patient registry or if disadvantages of the registry are named that the person themselves perceive.	Attitude_dPR_other	„Well, first of all (...) that's where I somehow come to the registry (laughs) because processing means saving and evaluating and the evaluations first of all concern me, so when I look at the sugar values together with my doctor or the lung function values or the long-term development of all these measurements, but real medical research is of course when you examine and link the data from many patients and (...) yes, there are of course also various risks

	Code	Description/meaning, incl. application of the code	Not to be confused with	Anchor example
				that, for example, somehow wrong conclusions are drawn or that generalized (clears throat) results are implemented for all patients although not for all patients. ...) yes, of course there are also various risks that you can think of, for example, that somehow wrong conclusions are drawn or that generalized (clears throat) results are implemented for all patients although they don't apply to everyone, so every patient is individual and has their own problems [...]“
11	Attitude_dPR_other [**Use only the subcobes**]	Attitudes and experiences are described that the person assumes are of value to strangers (other people). It also describes the advantages and disadvantages that the interviewee assumes others might find relevant.<br><br>It is not used when *only* reporting on own experiences regarding the registry.<br><br>**Nur die Subcodes dieses Codes werden angewendet.** *(**Note:** This main code can be summarized in the analysis with the subcodes.)*<br><br>***Note:** In the coding or analysis, attention should be paid to whether a clear distinction can be made between concerns and motives. This should be coded additionally if necessary.*		
11.1	Attitude_dPR_other_Pro	This subcode is used when the described attitudes and experiences that the person ascribes to others are positive about the digital patient registry or when benefits of the registry are mentioned that others might perceive.	Attitude_dPR_self	„Mhm (affirmative) so in our experience the approval rate is very high there are very few who reject it so I suspect that the vast majority see these opportunities as we also see an (clears throat) extremely high willingness in our circles to participate in studies at all (...) (M5, #00:42:36#) [...] and (laughs) so there is always a great willingness because you want to contribute to enabling the therapist to make progress in therapy yes (...)"

	Code	Description/meaning, incl. application of the code	Not to be confused with	Anchor example
11.2	Attitude_dPR_other_Con	This subcode is used when the described attitudes and experiences that the person attributes to others are negative towards the digital patient registry or when disadvantages of the registry are mentioned that others might perceive.	Attitude_dPR_self	„So I can imagine that someone might not give their consent (...) I mean maybe they're not sure what exactly happens to the data now. (4 sec.) Or whether they might not be used for something else, outside of research or something else, I don't know […]“<br><br>"(...) and risks if someone is suspicious of either the institution, there are of course also a few people who we may have disappointed as an association and who have a negative attitude towards us, or you have to trust the doctor too, of course, that he enters the right data, that he draws the right conclusions, that he discusses it with me if the relationship is disturbed, that can contribute to it or there are also very individual reasons that you don't necessarily have to be able to understand (laughs) […]“
12	InfoExchange	The interviewee describes how they were informed about the patient registry.		„There is an information sheet that is somehow four pages long or six pages (...) where all the important things are on it and then there are also links where you can see the data protection concept and the concept as a whole for the registry and also the reports we have the registry reports that are written for scientists and doctors and there is a parallel report for patients that is more readable (laughs) I say [I: Okay. (laughs)] and which we also always advertise among the members and point out via social media that it has been published again so that many patients know about it before they decide and also know about the benefits, so yes, but in answer to your question, there are about six pages of information with links to the Internet for further information and with a reference to this external site where you can also ask questions or [I: Okay.] complain (unintelligible)."<br><br>„I have to say that I don't remember it either [I: Okay.] So I don't remember when that was (...) and I remember, I just don't really remember it anymore. [I: And.] (...) So I think I remember I had to

	Code	Description/meaning, incl. application of the code	Not to be confused with	Anchor example
				sign something and (...) yes, the note also said what it was used for, but I can't remember anything specific.“
12.1	InfoExchange_Expectation	The interviewee describes what information they would have liked to receive in addition to the information they have already received.		„[…] but it might also make sense to put together a short written brochure on the subject, although this is always a problem with evidence-based brochures […]“
13	Measures_PO	The interviewee describes measures for the development, implementation and management or maintenance of a patient registry. These measures can be measures that are actually taken by a patient organization, or measures that the person would like a patient organization to take or design.		„So the registry must be scientifically well founded and then also well conducted, just like with studies, the approaches must first be correct in terms of the assumption. (...) Well, data protection must be in place. (...) Yes, [I: Yes.] (...) so (...) these are self-evident [I: Yes.] actually.“
Broad Consent				
14	BC_Pro	The interviewee describes the opportunities and advantages she sees in the introduction of broad consent for research studies and the handling of data.		„[…] So, I personally have no problem with this because I know that the data is processed anonymously and pseudonymously and, above all, that private users, i.e. pharmaceutical companies, only receive aggregated data, i.e. no data is passed on where any conclusions can be drawn about me and therefore I see this as unproblematic if a research project is considered worthy of funding by the association's committees and my data is also used there […]“
15	BC_Con	The interviewee describes the risks and disadvantages she sees in the introduction of broad consent for research studies and the handling of data.		„But then I just can't see through enough what's happening, so if you tell me I want to use your data for this and that project and I then also include the non-European data space with this and that goal, okay, but now to promise to release it for all eternity with an unknown goal, I actually still have inhibitions simply [I: Okay.] because I don't know enough what's happening, so I actually lack information to be able to make a good, informed decision there.“ (
16	BC_Measures	The interviewee describes which measures would increase trust when giving a broad consent or under which conditions they would give a broad consent.		„In general, of course, we would WISH to have such a (...), i.e. instead of a broad consent, one in which individual decisions can be made later, so we also discussed this in the German Ethics Council in the Big Data Opinion, that such a fiduciary data release could be made ne one could imagine that there is a patient panel with

	Code	Description/meaning, incl. application of the code	Not to be confused with	Anchor example
				patient representatives of this disease who then look at such a research project and are given the task of looking at it and if you agree then I am also then I also release my data for it or that would be the optimum with every research project I am contacted and asked if I agree […]“
17	Miscellaneous	This code can be used for text passages that the person coding them considers relevant to the research questions (see above), but which do not match any of the above codes. If necessary, new codes can be created from this during the coding process.<br><br>If several text passages are found for the same category (see the example here), a new code can be created.		*Financing and long-term maintenance of a registry:*<br>„So there is a standard, so to speak, for the operation of registries, so the data must be on a different server than the personal data, and consent and information must be given and this must also be renewed regularly and the person who is the sponsor, i.e. the one who finances it, must not be the one who looks at the data and so on and (...) these rules must be observed and the problem that we mainly see is that the financing. [I: Okay.] There is only one law in Germany for the [unintelligible] - Registry there is also a certain amount taken from the health insurance companies for the registry and it is publicly financed, but at a level I'll say what data is collected is of course at a much lower level than our registry now, but we have to invest in this quality assurance project, so everything that has to do with the registry and also with the projects that are connected to it, we spend half a million euros a year [I: Okay.] and finance it with donations. (...) And of course that's a problem for other rare diseases that are even rarer than ours (laughs) can't afford it [I: Yes.] and also other organizations - I think that's one of the main reasons why there aren't many registries for many diseases.“
Ethische Bewertungsdimensionen				
18		***Note**: The ethical evaluation dimensions are coded separately. In the analysis, these codes can be combined with the upper code codes in order to make more specific statements (e.g.: only for digital registries or specific digital technologies).*		

	Code	Description/meaning, incl. application of the code	Not to be confused with	Anchor example
		If necessary, it should be decided to code these codes in a separate coding step.		
18.1	ETH_Soli	**Solidarity**: The interviewee states that a reciprocal attitude with a reference group is important, or on the contrary not important, for the use of digital services. An important aspect of solidarity is direct or indirect support from the reference group or people in this reference group and their experiences, goals and activities.	ETH_Altr [An expression of solidarity should not be confused with altruism: Solidarity must have a reference group (in the background) as well as an expectation of reciprocity].	„[...] but maybe I can also help others who have my disease and say that we can then do research with this data in order to be able to help the general public, i.e. this general public of (K[1]) patients at some point in the near or distant future, depending on the case."
18.2	ETH_Altr	**Altruism**: The interviewee states that the use of digital services, especially the digital patient registry, is an expression of support or assistance to others, without expecting any benefits for themselves or anything in return from others (e.g. others in a reference group).<br><br>***Note****: With the definitions of solidarity and altruism, a theoretical decision has already been made. However, this seems sensible to me for the time being, because if we ultimately understand solidarity as a sub-code of altruism, we can take both codes together in the analysis.*	ETH_Soli [An expression of altruism should not be confused with solidarity: Altruism lacks the expectation of reciprocity, so there does not necessarily have to be a reference	„[...] And yes, if it's anonymous then it can actually only help. (...) If not for yourself then maybe for others or for the future or (...) I don't know if you can, so actually I think it can only help."

	Code	Description/meaning, incl. application of the code	Not to be confused with	Anchor example
			group (in the background)].	
18.3	ETH_Benefit	**Self-interest**: The interviewee states that the use of digital services, either by a PO or by an individual or PO member, is in their own interest - without reference to the interests or support of other people - out of hope for their own gain or advancement.		„ […] Hm, I thought that if my data is entered there (...) so that I enter it in the hope that it will either help me somehow by the fact that this data that applies to me will at some point apply to a drug where they say okay patient X Y with this data and other patients with this data can take it“
18.4	ETH_Just	**Justice**: The interviewee comments on whether there is (un)equal treatment in the context of digitalization or access to digital products (equal treatment), whether access and use of digital products are (un)fairly distributed or whether the treatment of people is fair.<br><br>An important aspect of Justice is the accessibility and usability of digital services for different groups of people.		*On justice of access:*<br>„[…] One aspect that comes up again and again in discussions in our self-help group is that there are many older people (...) who do not use the new aids. And we have to do something for them too. Yes? (...) But the consequence must not be that we say well then we won't do anything in the digital area because the elderly can't keep up [I: Okay yes.] (...) because from my point of view it can't be THAT at all. So if you do, then you have to double-track because at some point the older generation will have died out or will be fewer and fewer and if I only start using digital tools THEN it's actually too late. [I: Yes.] So you should set up the digital tools directly as far as possible (...) and offer an alternative as a backup for those who don't want to get involved.“<br><br>*On accessibility:*<br>„[…] yes, I was rather annoyed about the really badly made (...) website [I: Okay.] because it was a site that is used by visually impaired people and then the structure is extremely small and (...) yes, hardly manageable without magnification (laughs) [I: Recognize yes.] so those are the little things where I think ey that could have been designed a bit more friendly for the people concerned.“
18.5	ETH_Contr	**Data sovereignty** (control over one's own data): The interviewee comments on whether people have the		„That's important, so if I had the feeling that it's somehow not transparent or it's not clear who decides or or the employees in the

	Code	Description/meaning, incl. application of the code	Not to be confused with	Anchor example
		right to make decisions about their own health-related data. For example, they should always be able to decide for themselves which research projects use their own data.		office could look at the data themselves and consider (laughs) whether they are evaluating it now or something like that, then of course I would have my doubts or worries and would perhaps decide differently - that's a bit fictitious now because it's not the case […]"
18.6	ETH_DS	**Data security**: The interviewee comments on how information should be protected against unauthorized use, processing and loss (e.g. access, storage, manipulation and disclosure).		„[…] so that you can also look into these personal evaluations via an app or an iPad or something like that, but of course that's another level more difficult with data protection and data security no, you have to introduce an identification procedure, for example, so not everyone can come with an account and say I want to log in and register and link it to the e-mail, but you really have to make sure that it's THE one [I: Yes] who has also provided his data and that's complicated, of course."<br><br>„Of course, it would be bitter if employers or institutions had the opportunity to get in touch with me for whatever activities where they might value one hundred percent health and then possibly take a look at the patient file and see oops, there are huge risks lying dormant - no, we won't do that."
18.7	ETH_Trust	**Trust**: The interviewee comments on whether they can rely on the digital services or the players driving digitalization in the medical field. This is accompanied by the assumption that the digital services or other players do not conflict with their own expectations, interests or wishes.<br><br>This can also include the interviewee naming people and/or institutions that they (do not) trust/do not trust or that they consider to be (un)reliable.		„So I need the personal advice of my doctor who says that's okay for me now [I: Okay.] so that's basically how it works, a basic explanation like this and this is how it works and he worked on this and this is behind it and then it's okay or I have a trust in the institution so that the hospital already uses good instruments, tested instruments, evidence-based instruments and not some nonsense [I: Yes.] so yes, that's fundamental trust."<br><br>„Hm (...) so I think that's because you put your trust more or less in a machine (...) where you also know that there can be system errors or something and I think that if you put your trust in a person, you can still have more security for yourself than if you just put it in a

	Code	Description/meaning, incl. application of the code	Not to be confused with	Anchor example
				machine or artificial intelligence [I: Yes.] i.e. software because that's something you probably can't grasp and can't imagine in that way […]“
18.8	ETH_Trans	**Transparency**: The interviewee states that it is (not) important to be able to understand how digital products work or why other actors are acting, or that the goals of other actors are communicated openly and clearly.		„[…] I can go to the homepage of the (PO[3]) if I want to know more, it's very transparent, then I can see [I: Okay, yes] what the data is, what is done with the data, with the data there is an annual report of the registry, a scientific report that I can easily access there is a patient report in patient-friendly language with patient-friendly information that I can read (. ...) the registry, if there are important questions relating to the registry, this is discussed in the (abbreviation for K[1]) point info, i.e. in the in the in the in the newsletter I can contact the people in charge at the office if I have any doubts or questions - it's totally transparent […]“
18.9	ETH_Emp	**Empowerment**: The interviewee states that it is (not) important that the autonomy or self-determination of individuals or groups of individuals (such as communities) is increased so that they are able to represent their interests and needs in a self-determined and responsible manner and to participate in decision-making processes.		„[…] to be able to exchange ideas with people of your own kind, I'll say, or with people who have also experienced other things, something like that might also be a possibility for (...) socialization or maybe even if you're in a kind of hole after a bad crisis and can't get out of it, that you can then talk to people who have had a similar course of the illness via the patient registry so that you can be encouraged to talk to them about the patient about how to proceed, for example […]“
18. 10	ETH_Other	**Other**: If the interviewee addresses ethical evaluation dimensions that are not included in the above explanations, then these should be coded with the code "other". Examples could be: fair communication, autonomy, epistemic justice, diversity, etc.<br><br>If several text passages are found for the same category (see the example here), a new code can be created.		*Zum Beispiel „Verantwortung/Haftung“:*<br>„ So who is liable for the use of digital (...) applications if things go wrong? For example, if a diagnosis is NOT detected in time due to the fact that only telemedicine would be used. Just as a question. [I: Yes. (...) Who is liable if the photo sent in is not good enough to detect the little tiny black spot? […]“

This is an English translation of the original German coding guide

Coding guide for expert-interviews for the interview study "*Digitization projects in medical research and care*" (as at 13.06.2023)

Dr. Henk Jasper van Gils-Schmidt, B. Sc.
Department of Health Sciences, Faculty of Life Sciences
HAW Hamburg
Ulmenliet 20
21033 Hamburg

Definitions

Digitization: *Digitization refers to the conversion of analogue data and information into digital formats. In a broader sense, digitization also describes the transformation of social practices that have taken place in analogue or in person into forms in which these practices are mediated by digital applications.*

Patient organization: *Patient organizations represent the interests of people with disabilities or illnesses and are committed to improving the care of those affected and strengthening the skills of those affected in dealing with their illness or disability. Many patient organizations are also politically active, engage in national and international networking, support medical research and initiate their own research projects.*
Patient organizations are usually associations of patients and/or their relatives. They do not primarily pursue economic goals and are usually non-profit registered associations.

E-health: *E-health (short for electronic health) means "health based on electronic data processing" and is a generic term for digitalization in the medical field. E-health refers to the category of medical applications and measures that focus on the digital support or digital relocation of medical diagnostics, treatment and care. E-health products can be offered by private companies, associations and organizations as well as public healthcare providers.*
Examples of such products include electronic patient files, digital patient registries, telemedicine for communication between doctors and patients, online pharmacies, prescription apps, bloodless glucose measurement for diabetics and emergency call buttons for senior citizens.

M-health: *M-Health (short for mobile health) refers to a subset of e-health activities and measures in healthcare that are offered on mobile, wireless devices such as smartphones or tablets. The areas of application include determining vital signs such as blood sugar or body temperature (see remote monitoring) or motivational applications that remind people to take their medication, for example (see self-management of illness).*
Examples of M-Health products are: Corona-Warn App (the official tracking app by the German federal government), applications for monitoring vital functions (e.g. blood pressure monitors, blood glucose meters), wearables (e.g. fitness wristbands).

Health literacy: *Health literacy encompasses the knowledge, motivation and ability of people to find, understand, assess and apply relevant health information in everyday life, for example to make informed health-related decisions. Health literacy also refers to people's ability to find their way around the healthcare system, identify suitable services and facilities and successfully claim their own benefits.*

Digital literacy: *encompasses the knowledge, motivation and skills of people to participate in everyday digital life and to use, learn or work with and critically evaluate digital applications.*

Broad consent: *Broad consent is a type of consent form in which patients agree that their data may be used in future research projects without the need for renewed consent. The content and scope of these future research projects are not yet known at the time of consent. The broad consent should make it possible to reuse existing data for new research purposes without having to go through a lot of organizational effort. Broad consent should also make it easier for the people who give their consent to contribute to research and prevent them from being overburdened by constant new requests for consent. The broad consent model is therefore intended to relieve the burden on researchers and patients and save costs by reducing the administrative burden. One criticism of the broad consent model is that it can jeopardize the autonomy of the patients whose data is collected and processed.*

Questions

- In terms of ethical opportunities and risks, how do people (with extensive experience in patient advocacy and/or extensive expertise in patient participation in the medical field; from here on referred to as experts) evaluate digital services in the medical field? Is the assessment different in the context of research and care? And how do they perceive the evaluation from the members' perspective?
- How is the current regulation of the use of digital technologies and the use of personal data assessed?
- How is the role of patient organizations changing as a result of digitalization?

- How have members of patient organizations been involved in digitalization projects and what measures have been taken to increase trust in digital products?
- How is the role of patients changing as a result of digitalization?
- In which areas can patients be more actively involved in research (or digitalization projects)?

- How do experts assess broad consent in the context of research studies?

	Code	Description/meaning, incl. application of the code	Not to be confused with	Anchor example
**Digitization in general**				
1.	Experience_Dig	Experiences that the interviewee has had indirectly in their professional function in the respective organization or with digitalization projects and digital applications or their use. This refers to specific examples of the use and/or establishment of digital offerings.<br><br>*If an evaluative moment is included here, then it must also be coded with a subcode of "Attitude_Dig".*	Attitude_Dig	Exactly, we just talked about the website relaunch. That means that we have redesigned our entire website.<br><br>I have another honorary position, I'm on the board of a large patient organization at the federal level, and in this area I was actually able to have a personal influence on the digitization of the healthcare system, for example, in the federal government's digitization strategy, that of the Federal Ministry of Health, where I was also involved in various process steps, in a stakeholder workshop and in two surveys.
2.	Attitude_Dig	It describes attitudes specifically towards digitalization in medical research and/or care or the significance of this digitalization. In other words, this code has an evaluative aspect, which can be both pro and contra.<br><br>It is not used when *only* reporting on personal experiences or discussing digital products.<br><br>**Only the subcodes are assigned in this category.**<br><br>*(**Note:** This main code can be combined with the subcodes in the analysis).*<br><br>***Note**: This code can be subdivided later in the process, based on the reading of the transcripts (= inductive), if necessary according to the difference between "research" and "care" in further subcodes.*		

	Code	Description/meaning, incl. application of the code	Not to be confused with	Anchor example
2.1	Attitude_Dig_Pro	This subcode is used if the described attitude of the organization to which the interviewee belongs or of the person him/herself towards digitalization in the medical field and/or the use of digital technologies is **positive**. Specifically, for example, opportunities or possibilities of digital technologies can be named or described here.	Experience_Dig AND Attitude_Dig_Con	(...) Smart home devices, for example, so that you can really control all your household appliances at home by voice control at some point, which is a huge benefit.<br><br>So digitalization offers an incredible advantage here in particular, in terms of achieving a dynamic process and also gaining more transparency and also more safety for patients in the end, and in a comparatively short time. So this area of drug therapy safety is one that (incomprehensible), what are they called? APS-Aktionsbündnis Patientensicherheit (= Alliance for Patient Safety), that there are several thousand deaths every year due to the side effects of medication. And if you could prevent a large proportion of these through well-designed digital medication management, that would be great medical progress and not a major issue for the future.
2.2	Attitude_Dig_Con	This subcode is used if the described attitude of the organization to which the interviewee belongs or of the person him/herself towards digitalization in the medical field and/or the use of digital technologies is **negative**. Specifically, risks or challenges of digital technologies can be named or described here, for example.	Experience_Dig AND Attitude_Dig_Pro	(...) So basically, I would say that a major shortcoming from our point of view is that the entire digitalization process in the healthcare sector has been developed completely without the people affected, so to speak. In other words, those who have always been regarded as users, customers, affected parties, so to speak. However, it is exclusively service providers, payers, developers, Gematik, i.e. those who specify the company, those who practically specify the products of the telematics infrastructure. So far, the patient has not been included in the process, in the sense that he or she has been actively involved, that he or she, and in my view that is actually the most important thing.<br><br>That's why all the files that are now on the market are not really accessible. And around seventeen million German citizens with various disabilities are simply left out. And THAT, of course, cannot be the future of digitalized and hopefully more equitable and inclusive healthcare.

	Code	Description/meaning, incl. application of the code	Not to be confused with	Anchor example
3.	Protection	It addresses the protection of patients through the current regulation on the use of digital technologies and the use of personal data in the medical field. This means that this code has an evaluative moment, which can be both pro and contra.<br><br>**Only the subcodes are assigned in this category.**		
3.1	Protection_Pro	This subcode is used when the interviewee explains the advantages of the current regulation of the use of digital technologies and the use of personal data in the medical field for the protection of the patients concerned and a positive attitude is evident.	Protection_Con	As far as the telematics infrastructure products are concerned, i.e. prescription [and] electronic patient records, (...) what else do we have? (...) For the patient, these are the two big ones, now the electronic certificate of incapacity for work. (...) I would say that they are already pretty well secured in terms of protecting those affected.
3.2	Protection_Con	This subcode is used if the interviewee explains the disadvantages/risks of the current regulation of the use of digital technologies and the use of personal data in the medical field for the protection of patients affected and a negative attitude becomes apparent.	Protection_Pro	But this system also has its own problems. In this respect, when you say sufficiently protected. So when I go to the (...) online appointment booking, I find it a rather opaque system, because these appointment bookings are usually made via external providers. A classic example is Doctolib and their handling of data and data protection requirements is, to put it mildly, very opaque.
3.3	Protection_Wish	This subcode is used when the interviewee mentions specific aspects that could or should be improved with regard to the use of digital technologies and the processing of personal data in the medical field.		Well, starting with our members. Of course, as a small self-help association, it's difficult… but nonetheless educate people to protect their PCs with virus protection software. Starting with, in quotation marks, little things, I think that's where it starts for many people, protecting their data themselves for the first time, because we can't take over that, everyone is really responsible for ensuring that their PC is password-protected, for example, or that their smartphone, we would, perhaps as an example, in an online introduction, have the question about our cloud system, what we do about it (...), that the cloud itself is protected on users' devices.

	Code	Description/meaning, incl. application of the code	Not to be confused with	Anchor example
				[...] that you at least have the possibility to differentiate, so to speak, what I give to whom or which research I want to support and which not, so to speak, that you build in such possibilities in order to be able to realize self-determination by knowing or seeing what is done with my data and what is not, where it goes, what comes out of it and what information do I get back from it?
Digitization in PO				
4.	PO_Dig_Change	This code is used when the interviewee describes the changes they expect or have already observed in the patient organization as a result of digital technologies. Specific examples of changes to the services offered, the previous processes and the procedures in the organization can be mentioned.		[...] I would also be interested to see what things will look like in the next few years. But I think it simply can't be prevented that certain processes will become digitalized in the future. So it will become more and more permanent and (...), as I said, of course, email communication and chat programs are already established for communication. We have video conferencing, tools for example, tools for project planning. We actually now have tools for every requirement in the work context that are widely known and used. And I don't think that there will be any fewer of them.<br><br>So, when I look at it, how long does it take to become a really good patient representative for such a classic indication in the GBA (Federal Joint Committee)? Three, four, five years. So I think the system really needs resources at the moment to find one hundred, one hundred and fifty people. And of course that can also be an inspiration for self-help in its current form. (...) Perhaps these will also be completely different profiles of people. Younger, more diverse, etc., just to, (...) to be able to adequately represent the patient voice at this new level, i.e. at Gematik or at the BfArM in this whole DIGA, DIPA approval process, where we also have to get involved as patient representatives (...) and everything else that is now emerging in new digital care structures, i.e. also in quality

	Code	Description/meaning, incl. application of the code	Not to be confused with	Anchor example
				assurance, (...) in order to be able to adequately represent the patient voice everywhere.
5.	Prob_Member	Complaints and problems expressed by members in the context of the organization's involvement in digitalization projects to date are described.<br><br>**Only the subcategories are coded here.**<br>These can be summarized in the analysis.		
5.1	Prob_Member_ Aspects	The interviewee mentions or describes specific aspects and/or problems that were expressed by members of the organization during their organization's involvement in previous digitalization projects and of which they were made aware.<br><br>It is important to ensure that the problems or aspects relate to digitalization within the organization and not digitalization in the healthcare system in general.		We have just had the point that sometimes, or with some members, it can be observed that a general defensive attitude has developed over the years. And a first step would really be to bring the motivation to learn and to be patient.<br><br>What we often come across, for example, are websites or tools that use captchas and where, as a [non-K1], as a [non-K1] person, you have to select a film [a specific object] on an image in the box. For people who are [K1] or [K1], this is of course very difficult to almost impossible. (...) Of course, we often get feedback on this and of course we always have to make sure that we either adapt the systems we use accordingly (...) or explain them again.
5.2	Prob_Member_ Handling	The interviewee explains the specific activities that the organization they work for has undertaken to overcome the problems expressed by members in connection with digitalization projects.<br><br>It is important to ensure that the problems or aspects relate to digitalization within the organization and not digitalization in the healthcare system in general		The patient registry, for example, where members can register their illness and then be informed about new therapies, for example, if something comes up in research. And first of all, people have to enter a lot of data about themselves, which is of course very, very sensitive, very sensitive health data. And of course we 8as poets?) have to do the educational work in advance to ensure that this data is really protected, i.e. properly anonymized, pseudonymized, for

	Code	Description/meaning, incl. application of the code	Not to be confused with	Anchor example
				example, and in this respect, trust naturally plays a very, very important role.
6.	Inclusion_Memeber_Areas	This code is used when the interviewee mentions or describes specific examples and areas in which members of the organization were involved in the organization's digitalization processes and helped to shape them.	Measures_Trust	Exactly, yes, as I said earlier, it is important to us that we involve the members in the projects from the outset. That also means asking for opinions, carrying out surveys. We have already done this in advance. If, for example, we were to carry out these surveys and then not really incorporate the results into the process, i.e. say to the outside world, okay, we now have a test group of ten or twelve people here who are testing, they give us feedback and we don't take the feedback on board, for example, and implement it in the way that we at the office, for example, think would be right. Of course, in my opinion, that would also be a major breach of trust if we didn't respond to the feedback or requests from the members in a targeted manner, but instead simply imposed something from the top down and said okay, here's the new system, now you can work with it.
6.1	Inclusion_Member_Significance	This code is used when the interviewee describes how relevant they believe it is to shape the digitalization of the organization together with its members. The interviewee gives specific reasons for their assessment.		(...) Really, really important. I'm not just saying that out of the blue, but it's very important because in the end, of course, the members should also actively work with the new systems or the new website. In other words, if we don't involve the volunteers from the outset, it would be more of a top-down process. And if you did it that way, you would end up with a product, for example the website, that doesn't work.
7.	Measures_Trust	This code is used when the interviewee names and describes specific measures that the organization has taken to strengthen members' trust in digital technologies.	Inclusion_Memeber_Areas	(...) Yes, what comes to mind spontaneously is that we are increasingly trying to use tools or systems that run on (...) German or European servers, for example. We have, let's take Zoom as an example again, you can also set on Zoom which server the seminars are stored on, for example, or when we record seminars, on which server they are stored, that we run the whole thing on the German server, as an example, or that the people who register on our

	Code	Description/meaning, incl. application of the code	Not to be confused with	Anchor example
				website have their own, you could register on our website members area, that there is an automated procedure for assigning passwords. These are such, such, such small things, but, yes, they can establish trust with the members. That in any case.
7.1	Trust_Member	This code is used when the interviewee describes problems and/or situations, which they believe weaken members' trust in digital technologies.		But what I also have to say is that some people are actually (...) yes, they are against digitization for reasons that are not always understandable, but rather reject the digitization of from the outset, i.e. any processes, because of course many of them don't have the expertise and of course they realize, okay, something is changing in my everyday life and suddenly everything is going digital.
Role of patients in digitalization & research				
8.	Role_Pat	This code is used when the interviewee describes what changes they see for the role of patients as a result of the increased use of digital health applications or participation in e-health and digitalization initiatives such as digital patient registries, telehealth or monitoring via app.		Yes, well, I believe that digital skills absolutely have to grow in the sense of lifelong learning. Not everyone has and is able or willing to do this, but you also need a structural path for this. For example, there are these “Kundig” training courses from Hanover University of Applied Sciences and Arts. That's my first little digital driver's license and it's really a basic module where I would say that this kind of thing should be rolled out to the entire population and, with the help of self-help, the self-help contact points, the consumer protection organizations and so on, should also be rolled out across the country in order to duly emphasize the importance of this structural element for self-determination.<br><br>So that an awareness of our own design options grows relatively quickly. This is precisely the kind of shift that we are experiencing with the new digital possibilities, so that we as patients can help shape our therapies in a more self-determined way. That's how I would like to put it, there are also very different levels and in many areas it is still and or (...) the expertise of a doctor is absolutely necessary.

	Code	Description/meaning, incl. application of the code	Not to be confused with	Anchor example
9.	Participation_Research	The participation of patients in research projects / digitalization projects is addressed. The interviewee describes their perspective on the opportunities and challenges of participation. There is an evaluative element here, which can be both positive and negative.<br><br>**Only the subcodes are assigned in this category.**		
9.1	Participation_Research_Opportunities	Areas are identified in which the interviewee believes more active participation of patients in research/ digitalization projects is possible.		From my point of view. (...) Let me put it this way, ideally it would be possible to stipulate by law that patient participation is mandatory in the development of products and digital tools. That would be patient participation from the perspective of patient representation, so to speak. (...) That. That would be great, of course, because then it would be practically automated, you would always have patients involved in the co-development. Ultimately, however, this doesn't require a law, but rather those who are the developers (...) and also the payers and service providers. They would just have to do it in a certain way, in the sense that it's already possible to involve patients everywhere at any time.
9.2	Participation_Research_Risks	The interviewee names specific challenges that they perceive and expect with regard to the participation of patients in research/digitalization projects. Both risks/problems associated with participation and limitations to participation can be addressed here.		But especially in the research projects I have now experienced, in two research projects, (...) similar to what I am doing here now. Okay, well, there were also expert interviews where I was invited, sometimes on site, and then I asked, well, do I get this…? What about compensation for expenses? And then they said no, it's not possible, we don't have it, we haven't priced it into the project. And I don't mean in the sense of a professional payment, but at least the expenses in the sense of an expense allowance or loss of working hours are at least compensated. And that can be planned and priced in from the outset, because most research projects now also require patient participation in terms of the tender conditions and then it

	Code	Description/meaning, incl. application of the code	Not to be confused with	Anchor example
				must simply be common practice that a corresponding compensation with reimbursement of expenses etc. is somehow priced in.
Broad Consent				
10	BC_Pro	The interviewee describes the opportunities and advantages the organization sees in the introduction of broad consent for research studies and the handling of data.<br><br>***Note****: No internal or external attribution is intended for this code, as this question was often discussed rather briefly. If this does appear important in the coding, this distinction can be added.*		[...] I would say that with this broad consent, it would of course be a great advantage for the researchers to have access to more data than was previously possible. (...) As I see it, of course, every person, every patient, can of course decide for themselves whether or not they consent to this. (...) That's why I don't see it as a problem.
11	BC_Con	The interviewee describes what risks and disadvantages the organization sees in the introduction of broad consent for research studies and the handling of data.<br><br>***Note****: No internal or external attribution is intended for this code, as this question was often discussed rather briefly. If this does appear important in the coding, this distinction can be added.*		And (...) also this topic (broad consent?), […]. I take a critical view of this against the background of these ethical considerations. I don't know what will happen to me if I have a rare disease, which is certainly the case with my underlying disease, not myself, but others with my diagnosis. We then have a genetic marker and, in case of doubt, they can be identified very clearly with very little further information. And you can't necessarily predict today what that might mean for their employability in one, two or five years' time, when the data can be linked together even faster and even better.
12	Miscellaneous	This code can be used for text passages that the person coding them considers relevant to the research questions (see above), but which do not match any of the above codes. If necessary, new codes can be created from this during the coding process.		Legal situation: What will become of this in the future and with what resources and regulations it can then be backed up and subsequently controlled and sanctioned, is of course another matter, as these are the usual political processes.

	Code	Description/meaning, incl. application of the code	Not to be confused with	Anchor example
		If several text passages are found for the same category (see the example here), a new code can be created.		
12.1	Miscellaneous_ Responsibility	This code can be used for the text passages relating to the question of "responsibility" that are experienced as relevant by the person coding. If necessary, an independent code can be formed from this during the coding process.		And we've now seen, for example, that there were five hundred thousand users of the electronic patient file at the first attempt, which is not a lot, because it was inadequately regulated, for example. Gematik has said we are not responsible. The responsibility lies with the service providers, for example for accessibility. They couldn't do it, they didn't want to. That's why all the files that are now on the market are not really barrier-free.<br><br>So in this case, I really believe in a well-regulated, i.e. that there will be a clear process responsibility for this, that there will simply be several levels that are responsible, but with clear interfaces and people responsible here and there and also fallback solutions if things don't work out. In the case of the ePA and when it becomes a platform, it will provide an even greater quality advantage or enable usability in the first place.
Ethical evaluation dimensions				
13		***Note****: The ethical evaluation dimensions are coded separately. In the analysis, these codes can be combined with the upper code codes in order to make more specific statements (e.g.: only for digital registries or specific digital technologies).*<br>*If necessary, it should be decided to code these codes in a separate coding step.*		
13.1	ETH_Soli	**Solidarity**: The interviewee states that a reciprocal attitude with a reference group is important, or on the contrary not important, for the use of digital services. An important aspect of solidarity is direct or	ETH_Altr [An expression of solidarity	And it would also be a step towards not just seeing a patient collectively or somehow individually as an exploitable source of data in a colonial manner, but to have precisely this shift again, that patients are happy and voluntary, if they are informed, (...) to give

	Code	Description/meaning, incl. application of the code	Not to be confused with	Anchor example
		indirect support from the reference group or people in this reference group and their experiences, goals and activities.	should not be confused with altruism: Solidarity must have a reference group (in the background) as well as an expectation of reciprocity].	something back to the community with their data (...) and also help shape health innovations.
13.2	ETH_Altr	**<u>Altruism</u>**: The interviewee states that the use of digital services, especially the digital patient registry, is an expression of support or assistance to others, without expecting any benefits for themselves or anything in return from others (e.g. others in a reference group).<br><br>***Note***: *With the definitions of solidarity and altruism, a theoretical decision has already been made. However, this seems sensible to me for the time being, because if we ultimately understand solidarity as a sub-code of altruism, we can take both codes together in the analysis.*	ETH_Soli [An expression of altruism should not be confused with solidarity: Altruism lacks the expectation of reciprocity, so there does not necessarily have to be a reference group (in the background)].	But (...) also to raise this topic, such as this triage debate or assisted suicide or something like that, to a broader (...) social level. I think that's very important, because this data is really also (...) the raw material (...) with which we can essentially shape collective and individual health, how that, how good that can actually look for the individual or for the whole in the next five or ten or twenty years.
13.3	ETH_Benefit	**<u>Self-interest</u>**: The interviewee states that the use of digital services, either by a PO or by an individual or PO member, is in their own interest - without		(...) but rather that the person concerned benefits from it at all. So it has to. (...) I once said that if you introduce digital applications in the healthcare system, i.e. in the healthcare system or in care, then in my view they have to be financed with funds from the statutory

	Code	Description/meaning, incl. application of the code	Not to be confused with	Anchor example
		reference to the interests or support of other people - out of hope for their own gain or advancement.		health insurances, which is what they do, right? Then they have to generate added value or have a higher benefit than what is now realized in the previous analogue processes or analogue products.
13.4	ETH_Just	**Justice**: The interviewee comments on whether there is (un)equal treatment in the context of digitalization or access to digital products (equal treatment), whether access and use of digital products are (un)fairly distributed or whether the treatment of people is fair.<br><br>An important aspect of Justice is the accessibility and usability of digital services for different groups of people.		Gematik has said that we are not responsible. The responsibility lies with the service providers, for example for accessibility. They couldn't do it, they didn't want to. That's why all the files that are now on the market are not really accessible. And around seventeen million German citizens with various disabilities are simply left out. And of course, THAT can't be the future of digitalized and hopefully more equitable and inclusive healthcare.<br><br>Perhaps as an example, I think most people have become increasingly familiar with the fact that face-to-face events have been replaced by online events and video conferences since the start of corona. And what's important for us is that we also want to offer every event as an online event in future, because it's not always so easy for a person who [K1], for example, to travel from, I don't know, Cologne, Munich to Berlin for a training course or a seminar, which means barrier-free in the sense that the person can also participate online.
13.5	ETH_Contr	**Data sovereignty** (control over one's own data): The interviewee comments on whether people have the right to make decisions about their own health-related data. For example, they should always be able to decide for themselves which research projects use their own data.		Um, to (...) give the patient user even better control over where data actually flows off to (...) and for what purpose.<br><br>But on the other hand, there is a group of patients, especially in Germany with its historical past, who really want to say in a very self-determined way: Okay, I exercise the right that all my data actually belongs to me and that I can also determine where it goes in fine detail.
13.6	ETH_DS	**Data security**: The interviewee comments on how information should be protected against		But on the other hand. (...) there are also these (...) these problems of who will have access to this data in the future and where else it might flow and what it will be linked to [...].

	Code	Description/meaning, incl. application of the code	Not to be confused with	Anchor example
		unauthorized use, processing and loss (e.g. access, storage, manipulation and disclosure).		
13.7	ETH_Trust	**Trust**: The interviewee comments on whether they can rely on the digital services or the players driving digitalization in the medical field. This is accompanied by the assumption that the digital services or other players do not conflict with their own expectations, interests or wishes.<br><br>This can also include the interviewee naming people and/or institutions that they (do not) trust/do not trust or that they consider to be (un)reliable.		Oh, trust. (...) Yes, absolutely. Trust is a very big issue here, because of course, yes, every (...) tool, for example, also asks for data, so we're talking about data as a new currency, (...) which is of course also used profitably by many companies. So data is collected, used, anonymized or pseudonymized and passed on. (...) Accordingly, you have to go there and build up a certain amount of trust.<br><br>Everything else, including things like electronic medication plans, emergency data records, (e-prescription?) etc. So even the (e-prescription?) for the patient, the benefit is minimal. MINIMAL. Compared to what they had before. And that there. My. So the trust there. Okay, what does trust refer to, what does trust refer to would be, for example, data security or data protection or data security in the sense that the data is all transferred correctly , that no errors occur, that a mix-up doesn't happen during the operation, that I'm given the wrong medication etc.
13.8	ETH_Trans	**Transparency**: The interviewee states that it is (not) important to be able to understand how digital products work or why other actors are acting, or that the goals of other actors are communicated openly and clearly.		So this issue of transparency is a very important one for me, and of course it actually enables sovereign decision-making (...). And (...) that all Gematik applications, but also the others (...) can actually see in a patient-friendly way how far the data is released or who actually has the right to use what exactly for what purpose and for how long.<br><br>And by that I don't mean fifty euros per data set or anything like that, but that there should at least be transparency about (...) where exactly my data ends up in which research project, what is being researched with it and what comes out of it, where it is published and where I get the information, etc.

	Code	Description/meaning, incl. application of the code	Not to be confused with	Anchor example
13.9	ETH_Emp	**Empowerment**: The interviewee states that it is (not) important that the autonomy or self-determination of individuals or groups of individuals (such as communities) is increased so that they are able to represent their interests and needs in a self-determined and responsible manner and to participate in decision-making processes.		And (...) there are now also individual blossoms in the digital sector, they call themselves Patient for digital and the like, (...) where people with a service provider background have come together and are of course also trying to help shape (...) digital health. Who could blame them? Or initiatives such as Yes we can in our country, then out of their own concern. So I have to appreciate that. Absolutely. No question about it.<br><br>This app is not part of the professional association, but is being developed by a commercially oriented consortium. What does that tell us about the quality of this digital monitoring solution and its patient orientation? Well (...) as I know this and similar applications, (...) the monitoring aspect is out of balance with the empowerment aspect of the patients. (...) Among other things, because the design is the way it is and because patient representatives were only involved at a very late stage.
13.10	ETH_Other	**Other**: If the interviewee addresses ethical evaluation dimensions that are not included in the above explanations, then these should be coded with the code "other". Examples could be: fair communication, autonomy, epistemic justice, diversity, etc.<br><br>If several text passages are found for the same category (see the example here), a new code can be created.		But of course digitalization also has components that can very quickly lead to discrimination or even to sanctions and (...) yes, up to discriminatory withholding of therapies or aids or medical time. (Topic: Discrimination)
