# Supplementary material for: Critical consideration towards broad consent by patient experts: results of a semi-structured interview study on the secondary use of medical data
Source: BMC Med Ethics. 2025 Nov 18;26:163. doi: 10.1186/s12910-025-01326-x (PMC12629026; doi:10.1186/s12910-025-01326-x)
Supplement: Supplementary file 4 — Additional file 4. Quotes in the German original [file 12910_2025_1326_MOESM4_ESM.pdf]

Multimedia Appendix 4: Translated quotes		
**German Original**	**English Translation**	**Page**
"Das ist gut, dass man das machen kann und es wäre unethisch aus meiner Sicht, wenn man heute diese Daten nicht sammeln würde, um in der Zukunft Entscheidungen zu treffen und daraus ableiten zu können"	It's good that you can [use existing health data for research] and it would be unethical in my view if you didn't collect this data today in order to make decisions in the future and be able to derive decisions from it." (Representative_1)	p. 10
"Die einzige Möglichkeit, da was von zurückzugeben besteht darin, dass ich wenigstens dann, gerade bei so einer unbekannten Krankheit, bereit bin, mich zur Verfügung zu stellen, um zu versuchen, solange wie es nicht wehtut und so lange es mich nicht umbringt."	The only way I can give something back is that at least, especially with such an unknown illness, I am prepared to make myself available, as long as it doesn't hurt and as long as it doesn't kill me." (Representative_2)	p. 10
"Und im Kern finde ich auch, dass der Einzelne hinter der Gemeinschaft zurücktreten muss, sozusagen, weil letztendlich zählt die Gemeinschaft insgesamt oder die Gesellschaft und nicht der Einzelne im Wesentlichen"	"And in essence, I also think that the individual must take a back seat to the community, so to speak, because ultimately it is the community as a whole or society that counts and not the individual in essence." (Representative_3)	p. 10
"Vorteil ist eben, absolut für unsere seltenen Erkrankungen, die ja immer irgendwo hinten runterfallen. (...) Weil sie eben so selten sind, ist es ganz wichtig, da möglichst viele Daten möglichst aus dem breiten Gebiet zusammenzukriegen und nicht immer wieder von vorne eine Zustimmung zu erfragen, sondern, ja. Und letztendlich gerade die seltenen Erkrankungen. Es gibt ja immer noch nichts. Es gibt ja immer noch nicht, kein richtiges Basismedikament, es werden immer noch Symptome nur behandelt. Also. [I: Ja.] Es gibt ganz, also ganz viele wichtige Forschungsfragen, wo, die dann einfach auch den Ablauf und damit auch die Kosten verringern bei der Forschung."	"The advantage is, absolutely for our rare diseases, which always fall behind somehow. (...) Because they are so rare, it is very important to gather as much data as possible from the broad field and not to keep asking for consent again and again, but, yes. And finally, the rare diseases in particular. There is still nothing. There is still no real basic drug, still only symptoms are being treated. (…) There are many, many important research questions that simply reduce the process and therefore also the costs of research." (Representative_4)	p. 10-11
"Da muss ich ganz ehrlich sagen was sich auf (K[1]) bezieht würd ich meine Daten freigeben [I: Okay.] da hab ich überhaupt keine Probleme, (...so...) dass bessere Daten über die Versorgung entwickelt werden."	"I have to be honest and say that, if it is in relation to [the disease I have], I would release my data. (…) I have no problems with that at all, [so that] better data on care can be developed [...]." (Member_1)	p. 11

"(...) dass ich finde dass ein System etabliert werden soll, wenn man schon dauerhaft auch seine Daten hergeben soll, dass man auch was zurückbekommt. Und damit meine ich jetzt nicht irgendwie fünfzig Euro pro Datensatz oder so, sondern aber, dass zumindest eine Transparenz darüber hergestellt werden soll, (...) wohin denn meine Daten jetzt genau in welchem Forschungsprojekt eigentlich landen, was damit geforscht wird und was dabei, was dabei herauskommt, wo es publiziert wird und wo ich die Informationen krieg usw."	"[...] I think that a system should be established so that if you have to give up your data permanently, you get something in return. And I don't mean fifty euros per data set or anything like that, but that there should at least be transparency about (...) where exactly my data ends up in which research project, what research is done with it and what comes out of it, where it is published and where I get the information, etc." (Representative_3)	p. 11
"Ja das (...) das denke ich dass es wirklich schwierig ist denn (...) selbst wenn ich irgendein Papier unterschreiben also ich bin damit einverstanden dass meine Daten in dem und dem Rahmen benutzt werden und (...) einer Benutzung über diesen Rahmen hinaus (...) bin ich von vornherein nicht einverstanden oder einer zusätzlichen (...) Willenskundgebung sozusagen nur bereit [I: Ja.] dass das da ausgeweitet wird aber (...) letztendlich muss ich sagen (...) das das ist ja unkontrollierbar ne, das, das kann das kann der der Bürger X Y kann das ja letztlich überhaupt nicht kontrollieren ne."	"I think it's really difficult because (...) even if I sign some paper, so I agree to my data being used in this and that context and (...) I don't agree to any use beyond this context (...) from the outset or I am willing to an additional (....) expression of will, so to speak (…) that this will be extended but (...) in the end I have to say (...) that this is uncontrollable, right? The citizen XY cannot control this at all in the end, right?" (Member_2)	p. 11-12
"(…) aber jetzt zuzusagen in alle Ewigkeit freizugeben mit unbekanntem Ziel da hab ich tatsächlich auch noch Hemmungen einfach."	"but to promise to release it now for all eternity with an unknown destination - I'm simply actually still reluctant....“ (Member_1)	p. 12
"Weil ich, wenn ich die heute gebe, ja nicht sicher sein kann, dass die KI nicht in fünf Jahren das doch entschlüsseln kann. [I: Ja.] Wenn ich aber in fünf Jahren nochmal darüber reden darf, dann mache ich das vielleicht, weil heute gibt es vielleicht nicht die hundertprozentige Sicherheit, aber doch eine relativ große Sicherheit, dass es nicht passiert."	"Because if I give it today, I can't be sure that the AI won't be able to decode it in five years' time. (…) But if I'm allowed to talk about it again in five years' time, then maybe I will, because today there may not be 100% certainty, but there is a relatively high degree of certainty that it won't happen." (Representative_5)	p. 12

"aber andererseits ist es natürlich auch so, bestimmte Rahmenbedingungen ob es jetzt von außen ist von der Gesellschaft von der Sicherheit wie man die wahrnimmt oder wie auch immer können sich einfach auch ändern politische Lage können sich ändern es kann sich einfach auch über die Zeit die innere Einstellung zu gewissen Dingen verändern und (...) für meine Begriffe schwierig wenn man dann, ich, einfach mal ein Zeitfenster gegriffen jetzt für fünf Jahre sagt ja also hier ist eine breite Erlaubnis die Daten dafür und dort für dahin gehend zu verwenden"	"it is of course also the case that certain framework conditions, whether it is from the outside, from society, from security (…) can simply change, the political situation can change, the inner attitude to certain things can simply change over time and (...) from my point of view it is difficult if you then (…) simply take a time window for five years and say yes, so here is a broad permission to use the data for this and there for that." (Member_3)	p. 12
"also ja es wär vielleicht für mich wär es besser dann zu wissen, was genau von da geforscht wird sodass man na ja dass halt die Einwilligung dann doch für bestimmte Projekte immer wieder eingeholt wird [I: Okay.] (...) also von mir aus kann das auch gesammelt sein dass man mal dem einen zustimmt und einem anderen nicht ist natürlich wieder ein größerer Aufwand"	"[...] maybe it would be better for me to know what exactly is being researched so that (…) consent is obtained again and again for certain projects (...) so as far as I'm concerned, it can also be compiled so that you agree to one thing and not another is of course more work." (Member_4)	p. 13
"Also muss ich doch den Patienten entgegengehen und sagen, Pass mal auf, projektgebunden kannst du doch spenden, wo ist eigentlich dein Problem? Aber wenn man immer nur das große Ganze will, dann funktioniert das nicht. Vor allem nicht bei Patienten mit seltenen Erkrankungen. Denn noch dazu kommt ja hinzu, dass Patienten, die sehr schnell entdeckt werden können, auch in der Pseudonymisierung, Anonymisierung, erst recht darauf aufpassen müssen, was an ihren Daten rausgeht"	"So I have to meet the patients halfway and say, look, you can donate to a project, what's your problem? But if you only ever want the big picture, it doesn't work. Especially not for patients with rare diseases. Because on top of that, patients who can be detected very quickly, even in pseudonymization, anonymization, have to be even more careful about what of their data is send out." (Representative_5)	p. 13

"Aber ich denke, jetzt werden noch viele Leute zucken, wenn sie so eine, so ein broad consent an der Eingangstür in die Hand gedrückt bekommen. Und ich finde es auch nicht gut, weil wenn man da alles öffnet, dann kann man eigentlich gar nicht mehr den Patienten sinnvoll dazu beraten, wozu diese ganzen Daten denn genutzt werden und wie sie verknüpft werden und wer dann eigentlich darauf zugreifen kann und wer nicht."	"But I think a lot of people will cringe when they are handed one of these broad consents at the front door. And I don't think it's good either, because if you open everything up, then you can't actually give patients any meaningful advice on what all this data is used for and how it is linked and who can and can't access it." (Representative_1)	p. 13
"a, also wir stehen oder wir halten letztlich schon die informationelle Selbstbestimmung des Patienten hoch. (...) Das bedeutet in dem Zusammenhang, dass einerseits natürlich erst einmal so weit wie möglich aufgeklärt werden muss oder sollte, wofür die Einwilligung auch im Sinne eines breiten, breiten Einwilligung gegeben werden soll, welche Forschung damit betrieben wird. Also das ist immer die Voraussetzung. Ja, also eine wirksame Einwilligung hat man ja nur dann, wenn man eigentlich eine eine gesicherte Aufklärung vorgeschaltet hat, so. Das ist das eine. Und das andere, dass wir dafür eintreten, dass auf jeden Fall bei diesen Einwilligungen, der in dem Fall ja ist ja ein Patient meist oder der Bürger oder Bürgerin (...) ein ganzes Stück weit die Verwaltungshoheit darüber behalten soll"	"so we stand for or ultimately uphold the patient's informational self-determination. […] (...) In this context, this means that on the one hand, of course, as much information as possible must or should first be provided about what consent is to be given for in the sense of broad (…) consent, and what research is to be carried out with it. So that is always the prerequisite. Yes, you can only have effective consent if you have actually provided reliable information beforehand. That is one thing. And the other is that we advocate that in any case with these [broad] consents, which in this case is usually a patient or the citizen (...) should retain quite a bit of administrative sovereignty over it." (Representative_3)	p. 14
"weil ich zu wenig weiß was passiert da also da fehlen mir tatsächlich Informationen um da eine gute eine informierte Entscheidung treffen zu können."	"because I don't know enough about what's happening, so I actually lack information to be able to make a good, informed decision." (Member_1)	p. 14

"ob die Verbraucher*innen dann so eine mittlere Lösung Mittelgranulat wählt und sagt okay, drei, vier, fünf Knöpfe möchte ich aber selber regeln können. Und wenn du mich zwei oder dreimal gefragt hast, dann habe ich vielleicht auch durch die Nutzung dieser ganzen, was auch immer, digitalen Anwendung so viel Vertrauen und sehe, Es ist so viel Lebenserleichterung, dass ich dann auch sage okay, dann gehe ich da doch auf auch auch auf einen großzügigeren Konsens sozusagen. Und es gibt eben Verbraucher*innen, die haben ihre Gründe, das eben weiterhin auch alles ganz feingranular einstellen zu wollen. Das muss alles möglich sein, denke ich."	"whether the consumer chooses a semi-granular solution and says okay, but I want to be able to control three, four, five buttons myself. And if you've asked me two or three times, then maybe I have so much confidence from using this whole, whatever, digital application and see that it makes life so much easier that I say okay, then I'll go for a more generous consensus, so to speak. And there are consumers who have their reasons for wanting to continue to fine-tune everything. That must all be possible, I think." (Representative_6)	p. 14
"Vielleicht kann man sich darauf einigen, ein Broad Consent zu machen, aber dann bitte alle fünf Jahre erneuern. Revolving Consent nennt sich das. [I: Ja.] Interessanterweise habe ich das auch nie von Forschenden irgendwie gehört, dass sie es immer. Es ist alternativlos einen Broad Consent oder Informed Consent. Dazwischen gibt es nichts. Dieser Revolving Consent hat mehrere Vorteile, insbesondere den, wenn ich als Patient Angst habe, zu reidentifiziert wer_ reidentifiziert zu werden, so muss es heißen, dann ist natürlich, gebe ich meine Daten nicht."	"Perhaps we can agree to make a broad consent, but then please renew it every five years. That's called revolving consent. (…) Interestingly, I have never heard researchers say that they always do it: There is no alternative to broad consent or informed consent. There is nothing in between. This revolving consent has several advantages, especially that if I as a patient am afraid of being re-identified, then of course I won't give my data." (Representative_5)	p. 15
"Also wenn das wirklich gesichert ist dass das alles anonymisiert verläuft dann ja."	"So if it is really certain that everything is anonymous, then yes." (Member_5)	p. 15

"Aber solange wir dieses Verfahren haben, dass jemand eigentlich dazu aufgeklärt werden soll, was da passiert, ist das glaube ich nicht der richtige Ort und es ist nicht genug Zeit. Und jemanden mit, weiß ich nicht, irgendeiner schlechten Diagnose dann zu sagen, Naja, übrigens hier nebenbei, hier hast du noch so ein Stapel, das kannst du mal schnell unterschreiben, es ist gut für dich, das finde ich nicht in Ordnung. Also da sollte man schon mal drüber nachdenken und im Zweifel das auch mit nach Hause nehmen können und dann halt eine Entscheidung treffen, ob man das richtig findet, für die Zukunft seine Daten zu geben oder halt eben nicht."	"But as long as we have this procedure that someone should actually be informed about what's happening, I don't think that's the right place and there's not enough time. And then to say to someone with, I don't know, some bad diagnosis, well, by the way, here's a stack of papers that you can sign quickly, it's good for you, I don't think that's right. So you should think about it and, if in doubt, be able to take it home with you and then make a decision as to whether you think it's right to give your data for the future or not." (Representative_1)	p. 15
"Also einen kompletten Freibrief glaub ich würd ich nicht ausstellen aber ich glaube ich bin relativ bereit bei guter Information und sinnvoller Information schon frei also gewisserma_ also bestimmte Freibriefe auszustellen [I: Okay.] und darauf zu vertrauen dass die Wissenschaft das schon sinnvoll verwenden wird."	"So I don't think I would issue a complete carte blanche, but I think I am relatively willing to issue specific…. certain carte blanche if the information is good and useful (...) and to trust that science will use it sensibly." (Member_1)	p. 15
"Ja, also eine wirksame Einwilligung hat man ja nur dann, wenn man eigentlich eine eine gesicherte Aufklärung vorgeschaltet hat,"	"Yes, well, you only have effective consent if you've actually had a secure informed consent process beforehand." (Representative_3)	p. 16
"Ja also ich sag mal ich hätte ein Problem damit wenn jetzt meine gesamte Krankenakte der Forschung zur Verfügung gestellt wird ohne (...) dass man im Vorfeld (...) einen konkreten (...) Forschungsansatz hat also ich ich denke halt dass in dem Fall zu viele Daten in die Forschung abfließen würden ohne dass überhaupt ein Nutzen da ist"	"Well, I would have a problem if my entire medical file was made available for research without (...) having a concrete (...) research approach in advance, so I just think that too much data would flow into research in that case without there being any benefit at all." (Member_6)	p. 16
